# Hydrogen sulfide modulates actin-dependent auxin transport via regulating ABPs results in changing of root development in Arabidopsis

**DOI:** 10.1038/srep08251

**Published:** 2015-02-05

**Authors:** Honglei Jia, Yanfeng Hu, Tingting Fan, Jisheng Li

**Affiliations:** 1 College of Life Sciences, Northwest A&F University, Yangling, Shaanxi 712100, China; 2 Key Laboratory of Mollisols Agroecology, Northeast Institute of Geography and Agroecology, Chinese Academy of Sciences, Nangang District, Harbin 150000, China; 3 School of Biotechnology and Food Engineering, Hefei University of Technology, Hefei, Anhui 230009, China

## Abstract

Hydrogen sulfide (H_2_S) signaling has been considered a key regulator of plant developmental processes and defenses. In this study, we demonstrate that high levels of H_2_S inhibit auxin transport and lead to alterations in root system development. H_2_S inhibits auxin transport by altering the polar subcellular distribution of PIN proteins. The vesicle trafficking and distribution of the PIN proteins are an actin-dependent process. H_2_S changes the expression of several actin-binding proteins (ABPs) and decreases the occupancy percentage of F-actin bundles in the Arabidopsis roots. We observed the effects of H_2_S on F-actin in T-DNA insertion mutants of *cpa*, *cpb* and *prf3*, indicating that the effects of H_2_S on F-actin are partially removed in the mutant plants. Thus, these data imply that the ABPs act as downstream effectors of the H_2_S signal and thereby regulate the assembly and depolymerization of F-actin in root cells. Taken together, our data suggest that the existence of a tightly regulated intertwined signaling network between auxin, H_2_S and actin that controls root system development. In the proposed process, H_2_S plays an important role in modulating auxin transport by an actin-dependent method, which results in alterations in root development in Arabidopsis.

Auxin was the first identified phytohormone, and it plays a critical role in a variety of physiological and developmental processes in plants, including apical dominance, inflorescence and phyllotaxis development, embryogenesis, root development, lateral root (LR) and adventitious root initiation, tropism and vascular differentiation^1^. These processes depend on the establishment of auxin concentration gradients coordinated by auxin biosynthesis and polar auxin transport (PAT). Auxin is mainly synthesized in young aerial tissues and roots, particularly in the meristematic part of root apices^2^. From the auxin source in the shoot, plants transport indole acetic acid (IAA) to the basal tissues (e.g., the root) in a process that is dependent on the specialized PAT^3^ delivery system. In the root, PAT moves IAA to the root-shoot junction (basipetal) and towards the root apex (acropetal)^4^,^5^. Establishment of an auxin gradient with its maximum at the root apex is important for primary root growth and for LR development^3^,^6^.

PAT is mediated by auxin influx carriers from the AUXIN RESISTANT 1/LIKE-AUX1 (AUX1/LAX) protein family, and by auxin efflux proteins from the PIN-FORMED (PIN) family and the ATP-binding cassette protein B/multi-drug resistance/P-glycoprotein (ABCB/PGP/MDR) subfamily^7^. It has been reported that IAA is taken up into cells via AUX/LAX permeases through the ATP-dependent proton-driven anionic (IAA^−^) symport and MDR/PGP-type ABC transporters^8^,^9^,^10^. Recent studies have revealed that PINs regulate the auxin maxima in the distal domains of organ primordia^11^,^12^. Auxin efflux carrier PIN2 regulates the root gravitropism and the basipetal auxin transport in Arabidopsis roots^13^. In addition, the Arabidopsis *pin3pin4pin7* triple mutant shows reduced LRs^14^. Much data indicates that PINs are important regulators of PAT and root development^11^,^13^,^14^,^15^.

PAT is an actin-dependent process because the vesicle trafficking of PIN proteins requires the actin cytoskeleton^16^. A functional and intact actin cytoskeleton is necessary for root growth in Arabidopsis^6^. The actin cytoskeleton affects the subcellular dynamics of PIN proteins and impairs auxin efflux out of cells and auxin transport^17^. In response to these critical processes, the actin cytoskeleton needs to be rapidly and dramatically changed into different structures, and this organization is precisely controlled by various actin binding proteins (ABPs), including profilin, ADF/confilin, fimbrin, villin, formin, and myosin^18^. It is reported that ABPs are essential for the development of plant structures such as root hair^19^, primary root^20^, hypocotyl^21^, and pollen tubes^22^. In addition, the functions of ABPs were shown to be regulated by a variety of signaling molecules, including pH, PIP_2_, Ca^2+^ and PA^23^,^24^.

Hydrogen sulfide (H_2_S) is a colorless gas that has been considered toxic for many years. However, the biological effects of H_2_S have only been recently recognized. It seems that this gas is not only the agent potentially responsible for past mass extinctions but is also an important signaling molecule^25^,^26^. Furthermore, H_2_S was identified as the third endogenous gaseous transmitter, following the discovery of nitric oxide (NO) and carbon monoxide (CO)^27^. In plant systems, the mechanisms of H_2_S synthesis and release have been characterized for a long time^28^. However, very few studies have focused on the biology of H_2_S in plants. Only recently, the positive effects of H_2_S in response to the abiotic stress in plants, such as osmotic stress^29^, salt stress^30^, heat shock stress^31^ and heavy metal stress, have been studied^32^. H_2_S is also involved in the growth and development of plants. Many studies have shown that H_2_S is involved in stomatal closure, seed germination and increasing the growth rate^33^,^34^.

Recently, it has been reported that H_2_S can interact with IAA and can regulate the adventitious root and the LR formation in batatas and tomato plants^30^,^35^. However, the specific response mechanisms of the root development are less clear. In the present study, the interrelation between H_2_S and auxin on the root system growth was investigated. We provide evidence that H_2_S affects auxin distribution and transport by regulating the actin cytoskeleton in the Arabidopsis root.

## Results

### H_2_S inhibits primary root, LR and root hair elongation but promotes LR initiation in WT seedlings

**Figure 1:**
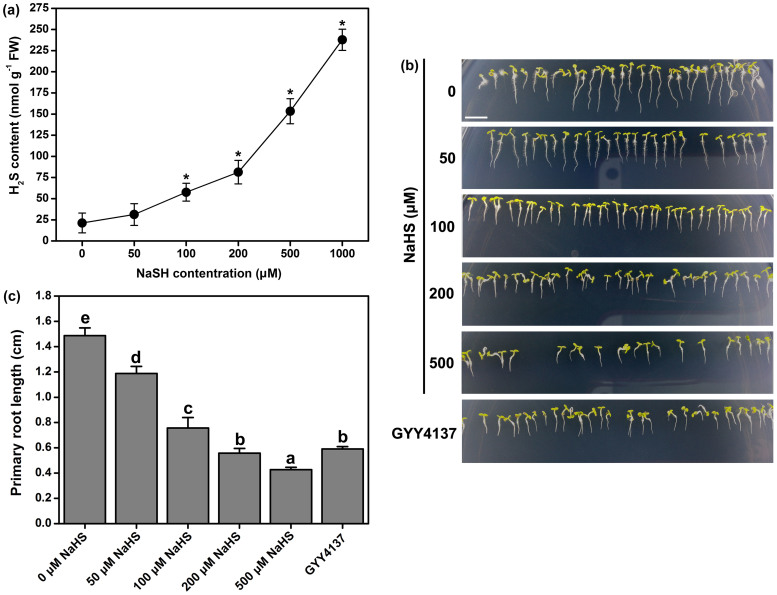
Effects of NaSH on endogenous H_2_S content in the root of WT Arabidopsis. (a) 5-d-old Arabidopsis seedlings were transferred to the 1/2 MS agar medium. Effects of NaSH on endogenous H_2_S content in Arabidopsis root. 50–1000 μM NaHS were used for various treatments for 6 h. (b) Effect of H_2_S in the regulation of primary root growth in WT Arabidopsis. Photograph showing the length of primary root of WT Arabidopsis seedlings. 3-d-old seedlings were transferred to 1/2 MS agar plates grown for 2 d. The agar plate were untreated (Control) or supplemented with 50 μM, 100 μM, 200 μM, or 500 μM of NaHS or supplemented with 200 μM GYY4137. Scale bar = 1 cm. (c) The length of primary root were obtained 2 d after the treatment of 3-d-old seedlings. Mean values and SE are calculated from three replicates in (a). Data are mean values and SE (n > 25) in (b) and (c). Within each set of experiments, bars with different letters are significantly different (*P* < 0.05, Duncan's multiple range tests).

It has been indicated that H_2_S is a secondary signal molecule that acts in response to the growth and development of plants^25^,^26^. The exogenous application of H_2_S donors was able to alter the endogenous H_2_S levels in a dose-dependent manner in Arabidopsis roots (Fig. 1a) and in other plants, such as maize and strawberry^21^,^29^. Changing the endogenous H_2_S levels can affect the root system architecture^36^. However, our knowledge of the molecular mechanisms by which way H_2_S regulates growth and development in Arabidopsis remains fragmentary.

To investigate the role of H_2_S in the regulation of primary root growth in Arabidopsis, WT (*Arabidopsis thaliana* ecotype Columbia-0, Col-0) plants were germinated on plates containing different concentrations of H_2_S released by the H_2_S donor - sodium hydrosulfide (NaHS) or GYY4137. As shown in Fig. 1, b & c, the inhibition was dose dependent, because a gradual decrease in the length of the primary root (from 1.5 ± 0.1 to 0.4 ± 0.1 cm) was observed as the NaHS levels increased from 0–500 μM. To validate our results by using other H_2_S donors, we tested GYY4137. The inhibition of primary root growth had a similar effect (59.8% inhibition in 100 μM GYY4137 treatment and 20.2%–72.1% inhibition under 50–500 μM NaHS treatments, respectively).

**Figure 2:**
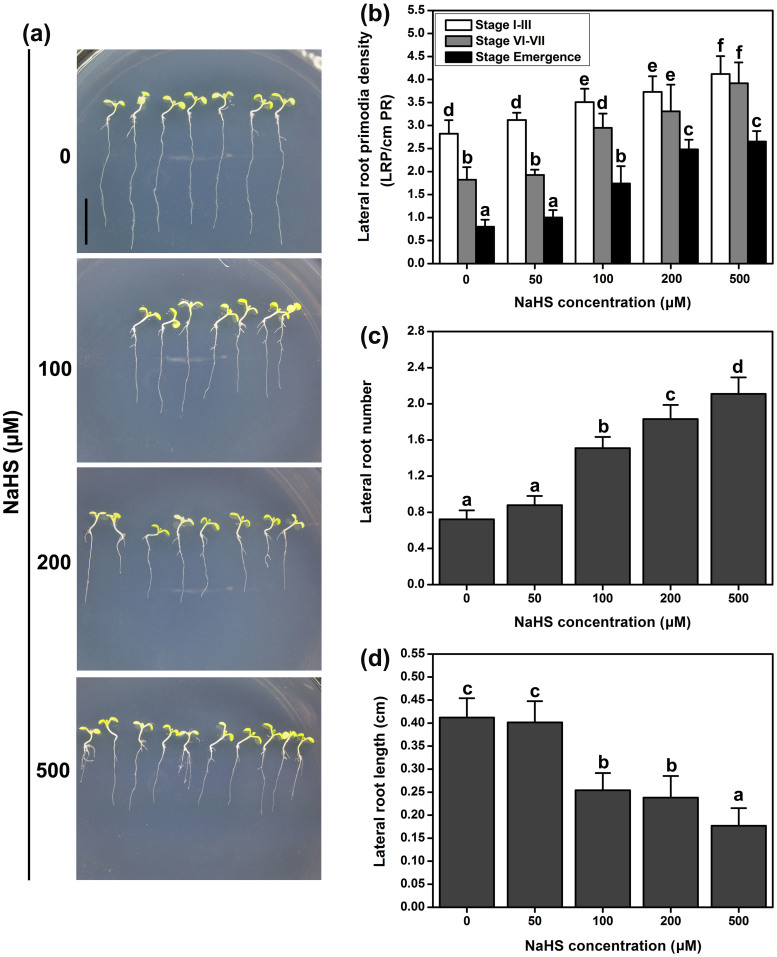
Effect of H_2_S on LR initiation and growth. (a) Effect of H_2_S in the regulation of LR growth in WT Arabidopsis. Photograph showed that 3-d-old seedlings were transferred to 1/2 MS agar plates grown for 4 d. (b) The density of LR primordia. Different stages of LRP (stages I–VII, emergence) were scored. 3-d-old WT seedlings were grown on agar plates supplied with 0–500 μM NaHS for 4 d. (c) The number of LP were obtained 4 d after the treatment of 3-d-old seedlings. (d) The length of LP were obtained 4 d after the treatment of 3-d-old seedlings. Data are mean values and SE (n > 25) in (b), (c) and (d). Within each set of experiments, bars with different letters are significantly different (*P* < 0.05, Duncan's multiple range tests).

LR and root hairs, which typically constitute the majority of the root systems in plants, contribute greatly to nutrient acquisition from soil; the development of these structures are regulated by many signaling molecules^37^. We examined the effect of H_2_S treatment on LR initiation and elongation. Considering these two developmental regions independently, we examined the effects of H_2_S on the lateral root primordium (LRP) and the LR, respectively. According to the anticlinal divisions and the cell expansion and periclinal divisions, the LRP development is grouped into I to III, IV to VII and emergence^38^. We observed an increase in the LRP initiation events when the 3-d-old seedlings were transferred onto NaHS. The density of the LRPs increased at 100 μM NaHS in all stages (I to III, IV to VII and emergence). At higher doses of NaHS, the number of LRP initiation events increased in a concentration-dependent manner (Fig. 2b). Thus, the effect of H_2_S on LRP initiation was not specific for any particular stage. An increased number of LRs were observed in the 3-d-old seedlings transferred onto NaHS at 4 d, but the length of the LR was inhibited by NaHS (Fig. 2c & d). These results indicate that the effects of H_2_S on the initiation and growth of the LR are different.

The root hair density and the root hair length dramatically decreased following treatment with 50 to 200 μM NaHS for 24 to 48 h (Fig. S1). The application of GYY4137 had the same effect as NaHS (Fig. S1). In addition, we found that when treated with NaHS, the germination time of the seeds was significantly shorter in contrast with the untreated seeds (Fig. S2). Together, the pharmacological data suggest that H_2_S plays an important role in Arabidopsis root system development, inhibiting the primary root, LR and root hair development while promoting LR initiation.

### H_2_S alters auxin response patterns and inhibits auxin transport in WT

**Figure 3:**
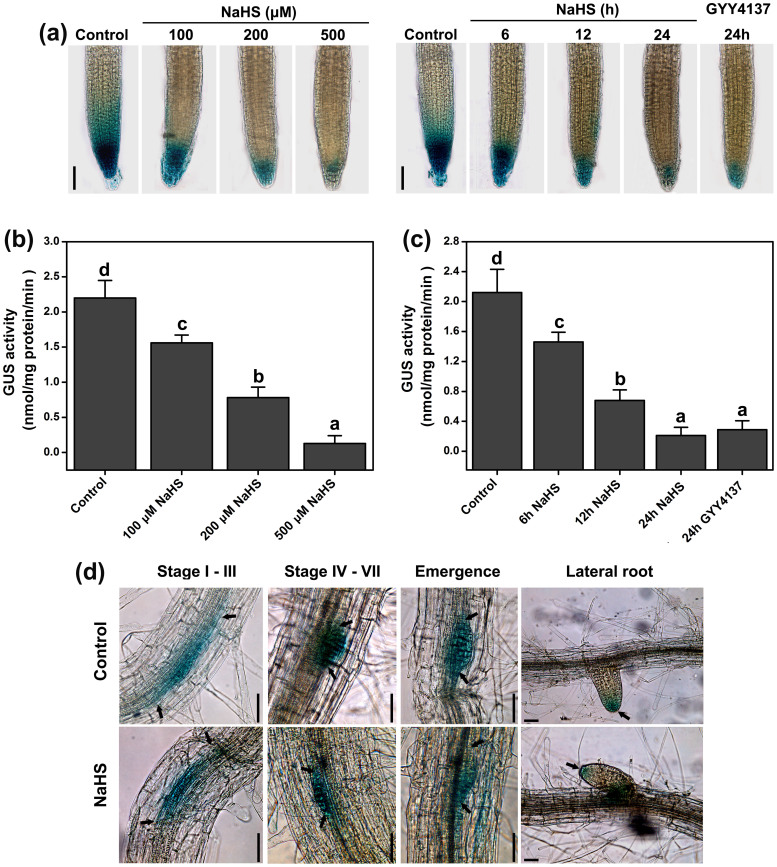
Effects of H_2_S on the auxin level of Arabidopsis root monitored by *DR5::GUS*. (a) Histochemical GUS staining patterns of *DR5::GUS* in 5-d-old seedlings treated with 100–500 μM NaHS for 12 h. Histochemical GUS staining patterns of *DR5::GUS* in 5-d-old seedlings treated with 200 μM NaHS and 100 μM GYY4137 for various time. Scale bar = 100 μm. (b) GUS activity of DR5::GUS in 5-d-old seedlings treated with 100–500 μM NaHS for 12 h. (c) GUS activity of DR5::GUS in 5-d-old seedlings treated with 200 μM NaHS and 100 μM GYY4137 for various time. (d) Expression pattern of *DR5::GUS* in LRP. 5-d-old Arabidopsis DR5::GUS seedlings were grown on agar plates supplied with solvent (Control) or 200 μM NaHS for 0–48 h. Images shown are representative of each treatment. Scale bar = 50 μm. Mean values and SE are calculated from three replicates in (b) and (c). Data are mean values and SE (n > 25) in (a) and (d). Within each set of experiments, bars with different letters are significantly different (*P* < 0.05, Duncan's multiple range tests).

Auxin response patterns based on auxin gradients are important factors in the regulation of many developmental processes, including cell division, elongation, and differentiation during primary root growth^6^. As an indicator of the auxin response, *DR5::GUS* is expressed in the root apex and during LR initiation^13^. *DR5::GUS* expression was assayed by histochemical staining and a quantitative assay of GUS activity. The control showed that *DR5::GUS* is expressed in the quiescent center (QC), columella initial cells and mature columella cells of the root apex (Fig. 3a). *DR5::GUS* expression could be attenuated in the three layers of columella cells and confined to the QC by increasing the H_2_S levels after application of NaHS (Fig. 3a). After treatment with 200 μM NaHS or 100 μM GYY4137 for 12 or 24 h, the expression of *DR5::GUS* gene was also markedly inhibited (Fig. 3a). To further validate our results, the GUS activity was quantified. As shown in Fig. 3b & c, the GUS activity decreased in a dose-dependent and time-dependent manner after NaHS application. In addition, GYY4137 had the same effect as NaHS on the GUS activity.

In Arabidopsis, LRP initiate exclusively from the pericycle founder cells, which are located opposite from xylem poles. Pericycle founder cells undergo several rounds of anticlinal divisions to create a single layered primordia composed of up to ten small cells of equal length (stage I). These cells divide periclinally, forming an inner and an outer layer (stage II). Further periclinal and anticlinal divisions set up a dome-shaped primordium (stages III–VII) that eventually emerge from the new LR (the emergence stage).

We next examined the distribution of auxin in several stages of LR development in the DR5::GUS transgenic seedlings following NaHS treatment. In the control seedlings, the site of LRP initiation (stages I–III) had the maximal *DR5::GUS* expression accumulation. A high level of *DR5::GUS* expression was observed in the next stages of LR development (stages IV–VII), which included the formation and emergence of the LRP (Fig. 3d). The results indicated that the entire developmental stage of LR required the maintenance of auxin distribution. In the NaHS-treated seedlings, no significant modifications in the *DR5::GUS* expression were observed over the stages of LR development (stages I–VII and emergence) compared to the untreated plants. However, the NaHS treatment markedly decreased the DR5::GUS activity in the apex of the mature LR (Fig. 3d).

**Figure 4:**
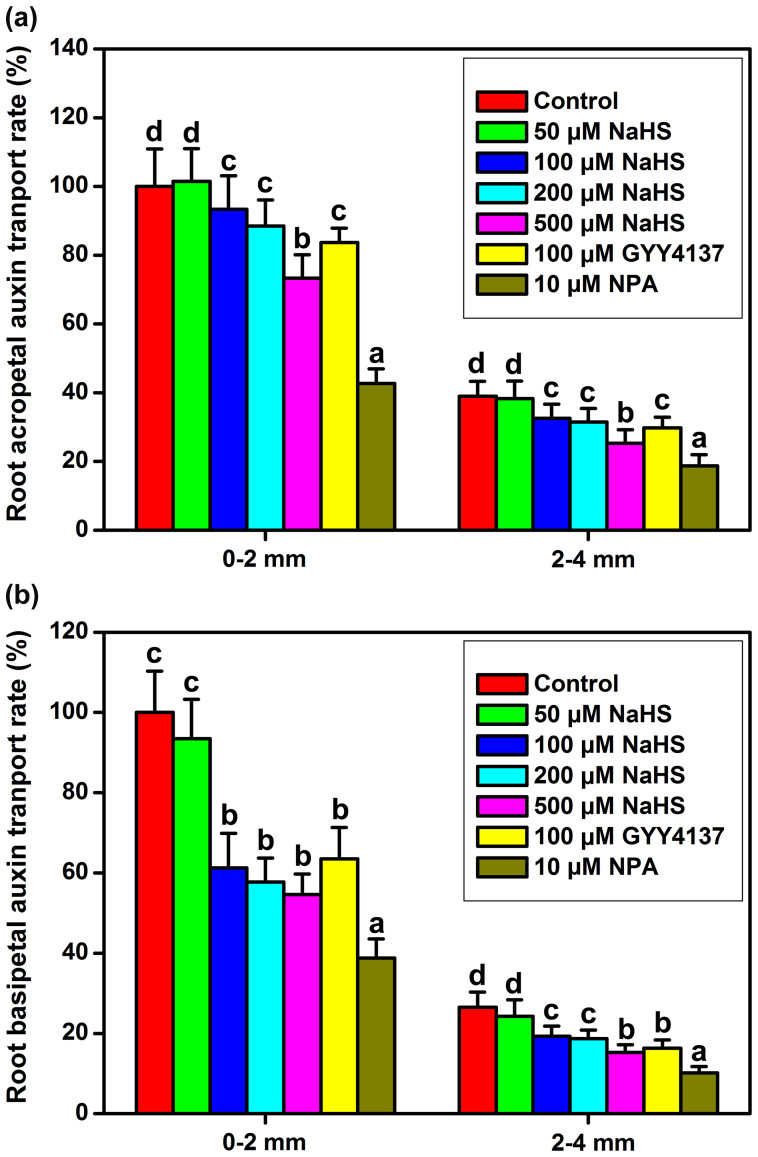
H_2_S regulates polar auxin transport in WT Arabidopsis root. Root acropetal auxin transport (a), and basipetal auxin transport (b) were assayed after various treatments for 12 h in 5-d-old seedlings. Mean values and SE are calculated from three replicates. Within each set of experiments, bars with different letters are significantly different (*P* < 0.05, Duncan's multiple range tests).

The observed change in the *DR5::GUS* expression pattern implies that auxin transport might be altered by increasing the endogenous H_2_S levels. To assess this hypothesis, we tested the acropetal and basipetal auxin transport in the roots of WT samples using [^3^H]-IAA. We used the untreated seedlings as the normalized samples and defined their value as 100% (Fig. 4). The normalized data showed that treatment with NaHS or GYY4137 markedly altered the auxin transport. A drastic reduction in acropetal and basipetal auxin movement was detected following NaHS and GYY4137 treatment (Fig. 4a & b). In addition, both the acropetal and basipetal auxin transport were decreased by NAP (Fig. 4a & b). These data supported the hypothesis that enhanced H_2_S levels cause a defect in the IAA transport capacity.

### H_2_S alters the subcellular localization of PIN1, PIN2, PIN4 and PIN7 in the root apices

**Figure 5:**
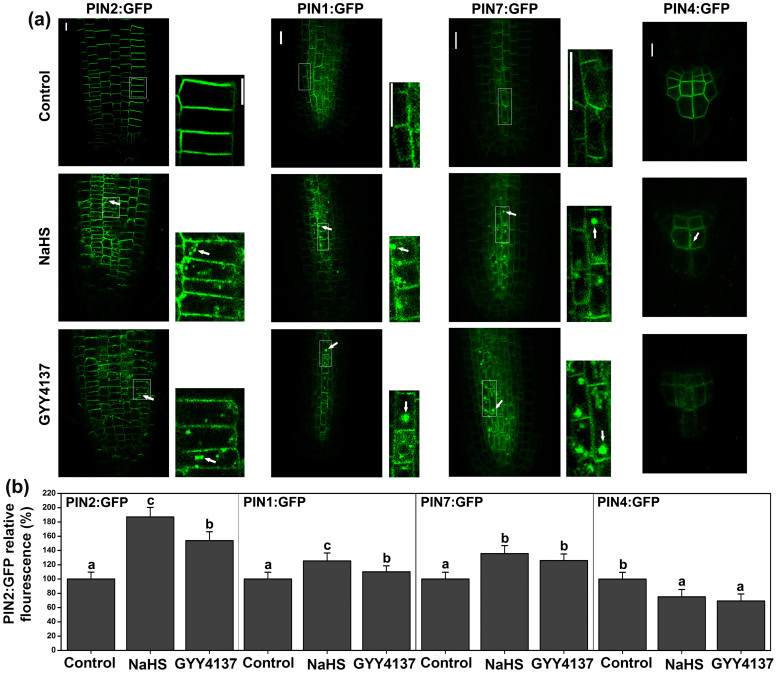
H_2_S modulates the expression and distribution of PIN proteins in apical zone of the primary root. (a) Distribution of PIN1:GFP, PIN2:GFP, PIN4:GFP and PIN7:GFP proteins were shown in untreated control plants, treated with 200 μM NaHS and treated with 100 μM GYY4137 for 6 h. Images shown are representative of each treatment. Scale bar = 5 μm. (b) Fluorescence density of PIN1:GFP, PIN2:GFP, PIN4:GFP and PIN7:GFP, the transgenic lines were treated with NaHS or GYY4137 for 6 h. Data are mean values and SE (n > 25) in (a) and (b). The arrows indicated the compartments formation of PINs. Within each set of experiments, bars with different letters are significantly different (*P* < 0.05, Duncan's multiple range tests).

The PIN proteins are important regulators in the establishment stage and in the auxin gradient in plants^11^. The polar subcellular localization of the PIN proteins at the plasma membrane determines the directionality of auxin flow^40^, thus contributing to regulation of multiple aspects of plant development^41^. Therefore, we examined the fluorescence of GFP fusions to PINs in Arabidopsis roots to determine if H_2_S could regulate the subcellular localization of the PIN proteins. The PIN proteins were clearly visible in the plasma membrane and showed a polar distribution in the control seedlings (Fig. 5). Treatment with NaHS or GYY4137 for 6 h showed the loss of their polar distribution at the plasma membrane in the root epidermal cells. Notably, a substantial amount of the PIN:GFP signal dissociated from the plasma membrane upon cytoplasmic entry (Fig. 5). In addition, the fluorescent intensity of PIN1:GFP, PIN2:GFP, and PIN7:GFP increased after the roots were treated with NaHS or GYY4137 for 6 h in root epidermal cells (PIN1 increased from 25.3% and 11.2% in response to NaHS and GYY4137, respectively; PIN2 increased from 87.2% and 53.9%; PIN7 increased from 35.6% and 25.9%, Fig. 5b). In contrast, the fluorescent intensity of PIN4:GFP decreased (by 24.7% and 30.6% after NaHS and GYY4137, respectively, Fig. 5b). The localization of PIN1 and PIN2 were observed in the *cpa*, *cpb*, *pfr3* mutants (Fig. S7). The plasma membrane localization of PIN1 and PIN2 was similar between the ABP mutants and the WT samples. After treatment with NaHS for 6 h, the PIN1 localization was altered in both the ABP and WT samples. In contrast, the localization of PIN2 also changed following 6 h NaHS treatment in the *cpa* and *pfr3* mutants and the WT samples. While the localization in the *cpb* mutant did not immediately change, after treatment with NaHS for 12 h, the PIN2 localization was altered in both ABP mutants and WT (Fig. S7).

qRT-PCR analysis of the expression of *PIN1*, *PIN2*, *PIN3*, *PIN4* and *PIN7* in the WT root showed that *PIN1*, *PIN2* and *PIN7* increased after treatment with NaHS for 3 to 6 h (Fig. S3). *PIN1* was recovered to the control levels by 6 h, and the expression of *PIN2* and *PIN7* decreased in 12 or 24 h (Fig. S3). The expression of *PIN4* decreased after treated with NaHS for 3 to 12 h, but recovered by 24 h (Fig. S3). However, the expression of *PIN3* did not show obvious changes after treatment with NaHS (Fig. S3). These experiments confirmed that H_2_S affects the localization of the PIN proteins.

### The effects of H_2_S on F-actin and APBs in the Arabidopsis root

**Figure 6:**
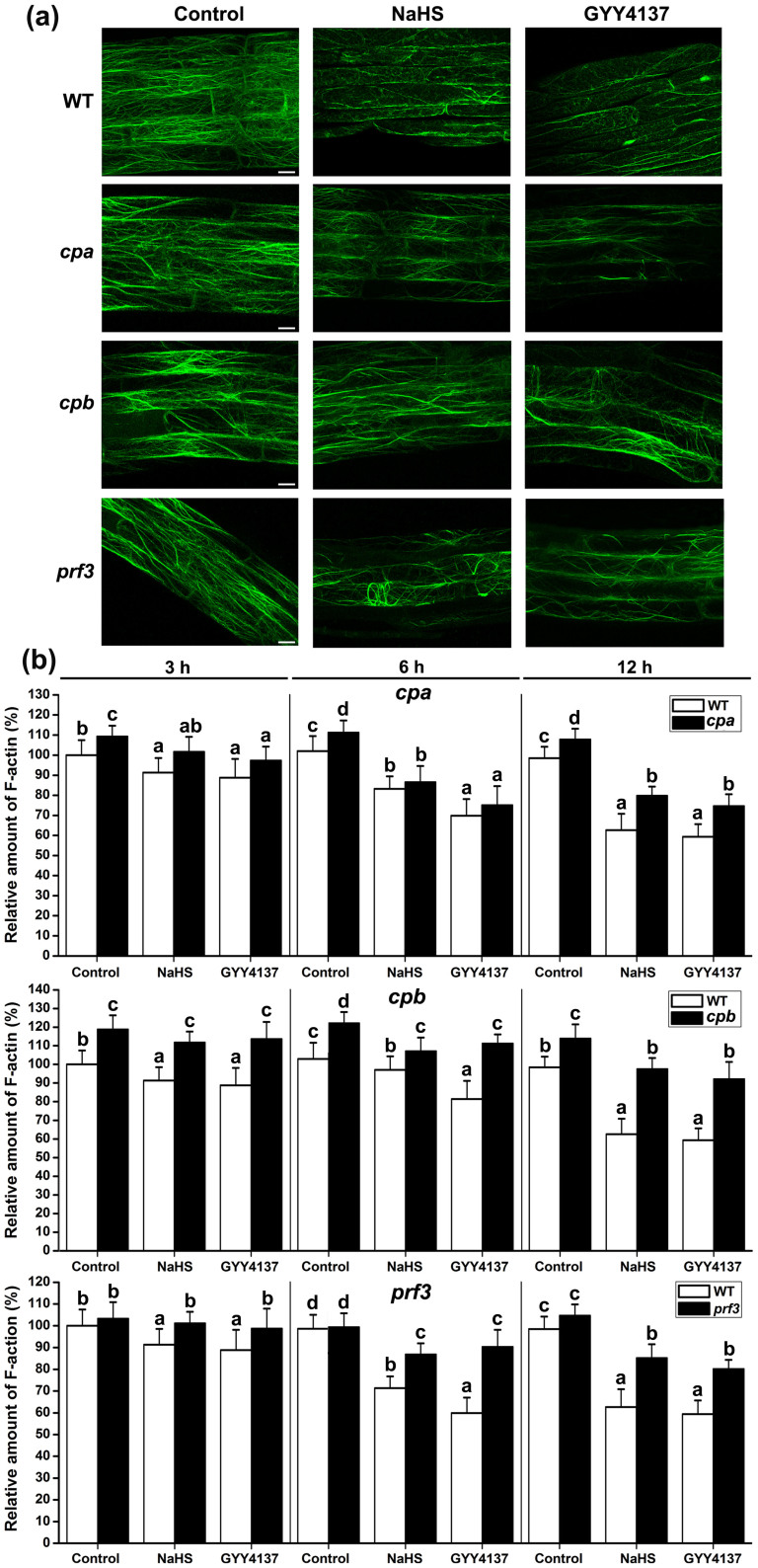
Effects of H_2_S on F-actin in root of WT, *cpa*, *capb* and *prf3*. (a) Distribution of F-actin was shown in untreated control plants, treated with 200 μM NaHS or with 100 μM GYY4137 for 6 h. Images shown are representative of each treatment. Scale bar = 10 μm. (b) Quantification of the relative F-actin levels. The amount of F-actin in untreated WT roots was normalized to 100% as the control. Data are mean values and SE (n > 25) in (a) and (b). Within each set of experiments, bars with different letters are significantly different (*P* < 0.05, Duncan's multiple range tests).

The actin cytoskeleton in eukaryotic cells is a highly organized and dynamic structure that plays a central role in numerous cellular processes, including intracellular transport, cell growth, and organelle positioning^42^. F-actin is known to affect the plasma membrane localization of PIN proteins, as the vesicle transport of PIN proteins depends on F-actin^43^,^44^. We examined the effects of H_2_S on the subcellular localization of F-actin (stained with fluorescein phalloidin), and the actin network in root epidermal cells observed by confocal microscopy. As shown in Fig. 6a, we observed that the elongation zone of the root tip and the actin network displayed thick, often longitudinally oriented cables and finer, randomly arranged filaments in the WT control. Treatment with NaHS or GYY4137 for 6 h caused significant changes in the actin cytoskeletal organization. After treatment, the thick actin cables were absent and the percentage of occupancy of F-actin bundles decreased in each cell (Fig. 6a).

**Figure 7:**
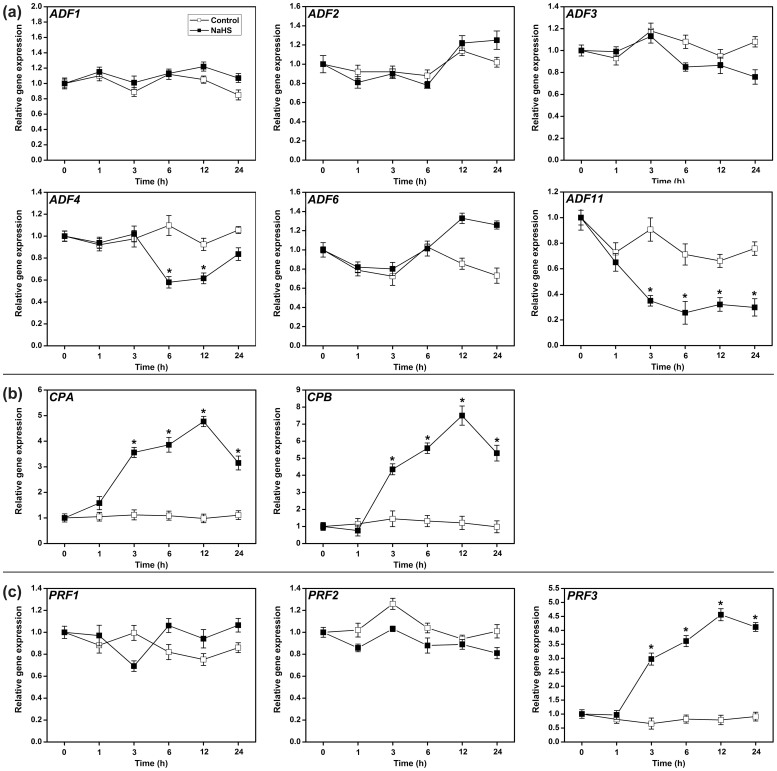
qRT-PCR analysis of ABPs genes in WT Arabidopsis root. Relative expression levels are normalized with an internal standard *EF1a*. 5-d-old Arabidopsis WT seedlings were grown on agar plates supplied with 200 μM NaHS for 1–24 h. Mean values and SE are calculated from three replicates. Within each set of experiments, bars with different letters are significantly different (*P* < 0.05, Duncan's multiple range tests).

**Figure 8:**
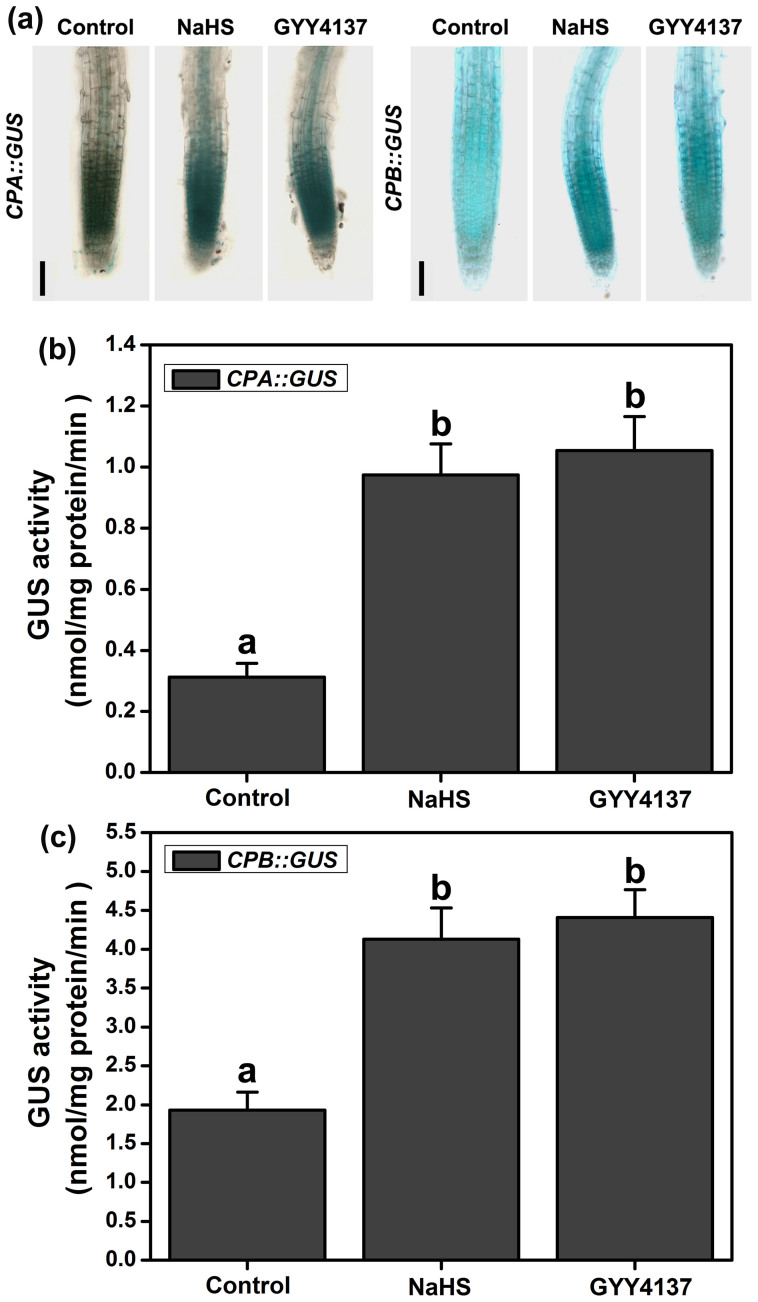
Effects of H_2_S on the expression of *CPA::GUS* and *CPB::GUS* in the roots of the transgenic lines. (a) Histochemical GUS staining patterns of *CPA::GUS* and *CPB::GUS* in 5-d-old seedlings treated with 200 μM NaHS or 100 μM GYY4137 for 6 h. Images shown are representative of each treatment. Scale bar = 100 μm. (b) GUS activity of *CPA::GUS* and *CPB::GUS* in the roots of 5-d-old seedlings treated with 200 μM NaHS or 100 μM GYY4137 for 6 h. Data are mean values and SE (n > 25) in (a). Mean values and SE are calculated from three replicates in (b) and (c). Within each set of experiments, bars with different letters are significantly different (*P* < 0.05, Duncan's multiple range tests).

**Figure 9:**
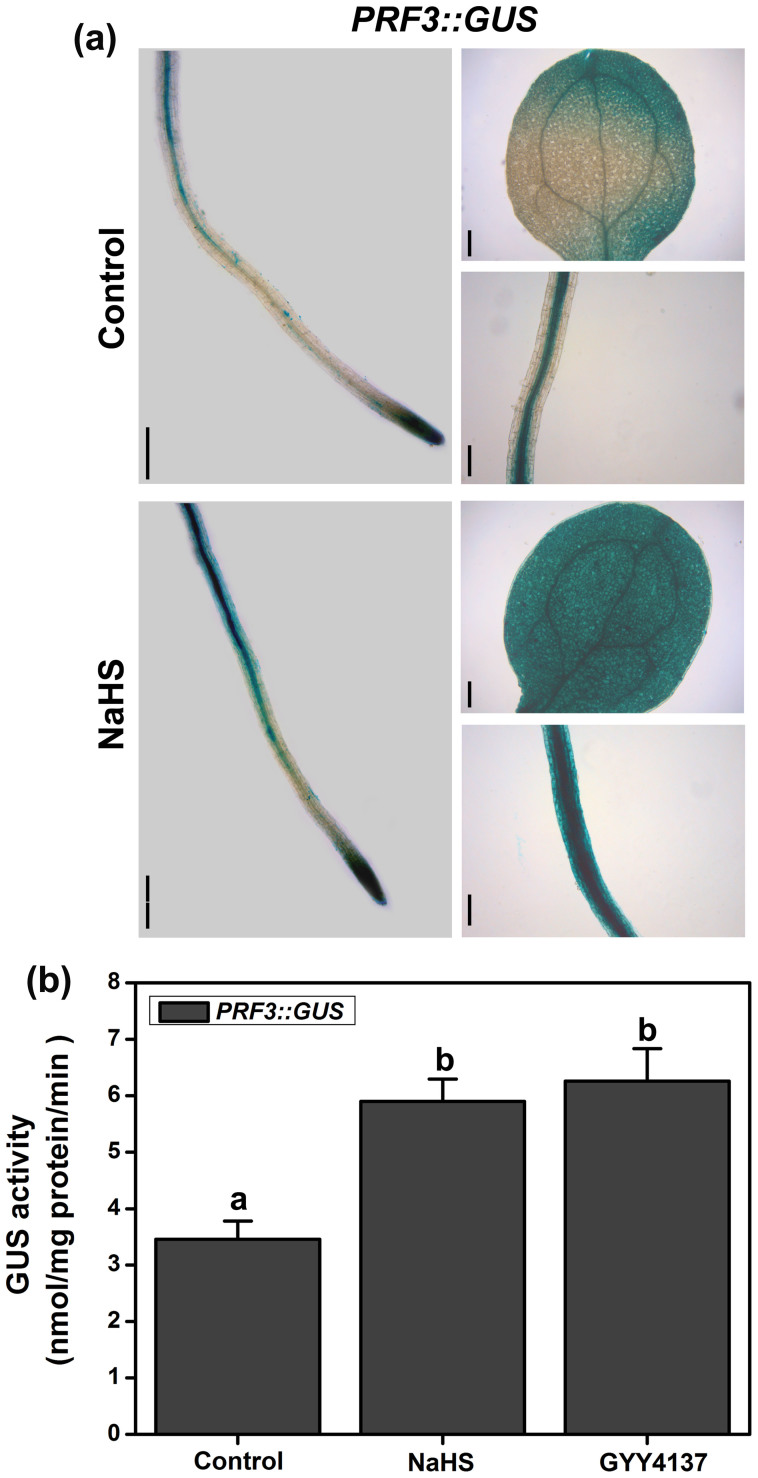
Effects of H_2_S on the expression of *PRF3::GUS* in the transgenic lines. (a) Histochemical GUS staining patterns of *PRF3::GUS* in 5-d-old seedlings treated with 200 μM NaHS for 6 h. Images shown are representative of each treatment. Scale bar = 200 μm. (b) GUS activity of *PRF3::GUS* in the root of 5-d-old seedlings treated with 200 μM NaHS or 100 μM GYY4137 for 6 h. Data are mean values and SE (n > 25) in (c). Mean values and SE are calculated from three replicates in (b). Within each set of experiments, bars with different letters are significantly different (*P* < 0.05, Duncan's multiple range tests).

A regulatory system that contains the actin filaments (F-actin) and actin-binding proteins (ABPs) is required for these processes, and the diverse actin cytoskeleton is directly controlled by different ABPs^22^,^45^,^46^. Actin depolymerizing factors (ADFs) sever F-actin and increase the rate of dissociation of actin monomers from the pointed ends^47^. Capping proteins (CPs) inhibit the growth of F-actin at the barbed end^23^. Profilins (PRFs) bind to G-actin to inhibit polymerization^48^. These proteins all have the potential to decrease the occupancy percentage of the F-actin bundles^20^,^21^,^49^. We examined the effects of H_2_S on the expression of *ADFs*, *CPs* and *PRFs*. qRT-PCR analysis showed that the expression of *ADF1* and *ADF4* decreased following NaHS treatment for 6 to 24 h (Fig. 7a), and the expression of *CPA*, *CPB* and *PRF3* increased after NaHS treatment in 3 to 24 h (Fig. 7b & c). We further examined the expression of *CPA::GUS*, *CPB::GUS* and *PRF3::GUS*. The results showed that the application of NaHS or GYY4137 significantly promoted *CPA::GUS* and *CPB::GUS* gene expression in the root apices (Fig. 8a). The *GUS* gene expression was also enhanced by NaHS in root and leaf samples of *PRF3::GUS* (Fig. 9a). The GUS activity showed a similar profile in the CPA::GUS, CPB::GUS and PRF3::GUS transgenic lines (Fig. 8b, c, & 9b). These data suggested that H_2_S may affect F-action via regulating *CPA*, *CPB* and *PRF3* expression.

To validate this hypothesis, we observed the effects of H_2_S on F-actin in T-DNA insertion mutants *cpa* (*CP α-subunit (CPA) mutant*), *cpb* (*CP β-subunit (CPA) mutant*) and *prf3*. As shown in Fig. 6, the percentage of occupancy of the F-actin bundles appeared to be somewhat more dense in the *cpa* and *cpb* cells than that in the WT cells. There were obvious differences in *cpa* and *cpb* compared with the WT cells after treatment with NaHS or GYY4137 for 3 to 12 h. The effects of NaHS and GYY4137 were partially removed in *cpa* and *cpb* (Fig. 6b). The *prf3* seedlings had similar amounts of F-actin bundles as the WT seedlings (Fig. 6b). However, the effects of NaHS and GYY4137 were also inhibited in the *prf3* seedlings compared with the WT seedlings in 3 to 12 h (Fig. 6b). These data imply that knockout *CPA*, *CPB* or *PRF3* could partially remove the effects of H_2_S on the percentage of occupancy of the F-actin bundles.

### The effects of H_2_S on auxin transport in the root of cpa, cpb and prf3 mutant seedlings

**Figure 10:**
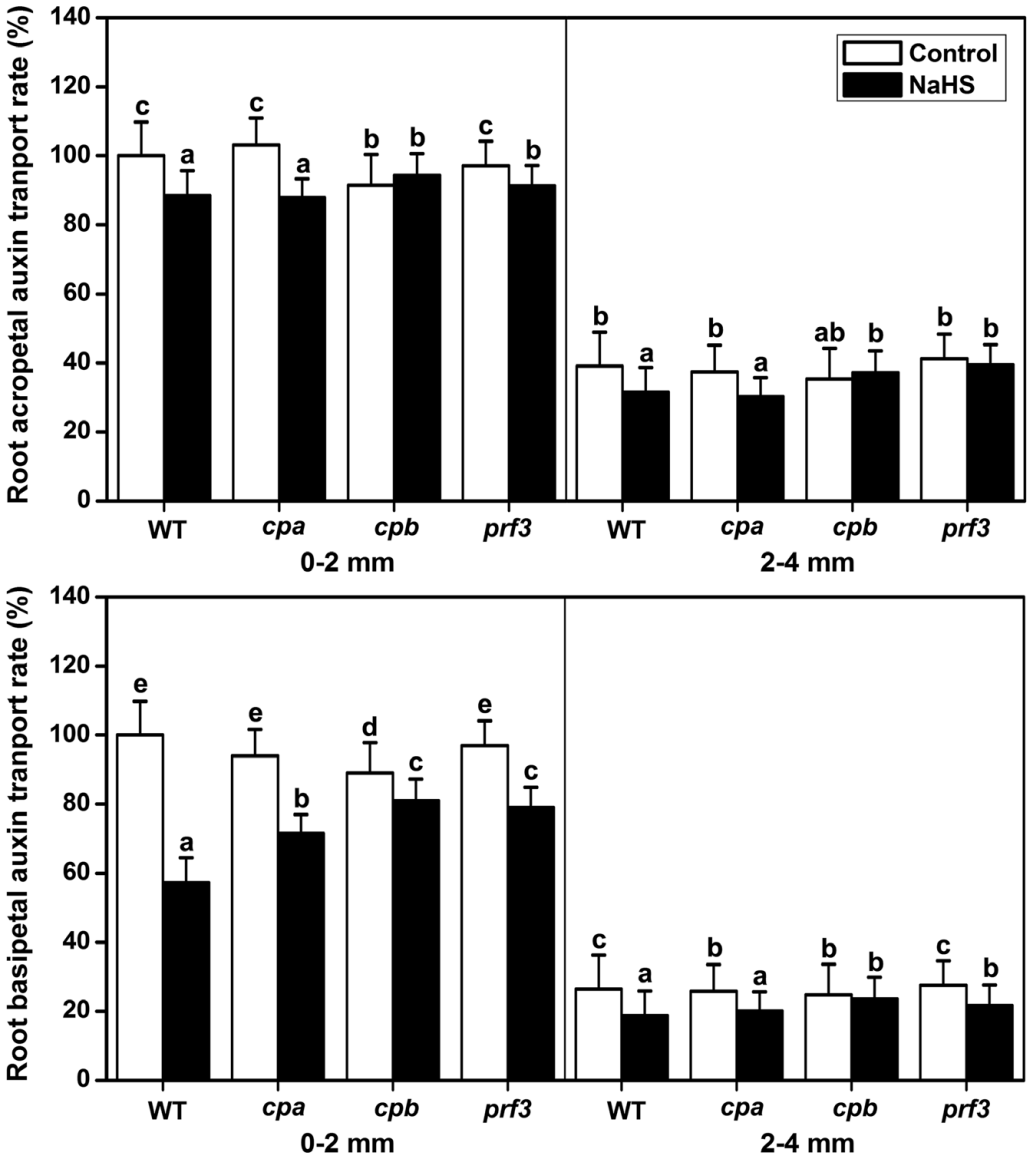
H_2_S modulates polar auxin transport in root of WT, *cpa*, *capb* and *prf3*. Root acropetal auxin transport (a), and basipetal auxin transport (b) were assayed after NaHS treatments for 12 h in 5-d-old seedlings. Mean values and SE are calculated from three replicates. Within each set of experiments, bars with different letters are significantly different (*P* < 0.05, Duncan's multiple range tests).

The acropetal and basipetal auxin transport rates were decreased in the *cpb* compared to the WT root samples (Fig. 10). In the roots of *cpa*, the basipetal auxin transport rates were decreased in 2 to 4 mm (Fig. 10). *prf3* displayed a similar auxin transport capacity as WT (Fig. 10). H_2_S could observably decrease the auxin transport rates in the WT sample (Fig. 4). Interestingly, there was an obvious difference between the mutant and WT plants after treatment with NaHS for 12 h. In the root of *cpb*, the effect of NaHS was removed in the acropetal auxin transport in both 0 to 2 mm and 2 to 4 mm, and in the basipetal auxin transport in 2 to 4 mm (Fig. 10). The weak auxin transport capacity was partially recovered compared with the WT levels in *cpa* and *prf3* after NaHS treatment for 12 h (Fig. 10). The H_2_S-induced inhibition of the primary root length was markedly removed in the *cpa*, *capb* and *prf3* mutant plants under 100 μM NaHS treatment and partly removed in *capb* and *prf3* under 200 μM NaHS (Fig. S4 and S5). These data suggest that CPA, CPB and PRF3 play important roles in H_2_S by regulating auxin transport.

## Discussion

The understanding of the importance of H_2_S as a regulator of plant growth and response to environmental stress has increased considerably despite the limited available information on its signaling. A physiological role for H_2_S in the regulation of root growth has been described^30^. H_2_S promotes adventitious root formation in batatas^35^ and LR development in tomato plants^36^. Much of the work with H_2_S signaling has involved the pharmacological application of H_2_S donors such as NaHS to emulate H_2_S production^15^,^50^. Using different H_2_S donors (NaHS and GYY4137), we confirmed that increased endogenous H_2_S levels inhibited primary root and root hair growth but promoted LR formation in Arabidopsis (Fig. 1, Fig. S1 & S2). However, the mechanisms of the precise cellular responses to H_2_S are not yet well understood. Therefore, the discovery of the novel signaling molecule H_2_S, which is involved in triggering root system architecture, was an important outcome of this work.

The process of root organogenesis is controlled by auxin^51^. Previous studies have demonstrated that H_2_S interacts with auxin in modulating root system growth^35^,^36^. Using *DR5::GUS*, an indirect indicator of the level and distribution of auxin, we observed that high concentrations of endogenous H_2_S attenuate auxin-dependent reporter expression in the QC and in the three layers of the columella cells (Fig. 3a). The maximum auxin distribution is found in the root apex, which is necessary for meristem maintenance^52^. The maximum levels are normally observed in the QC, columella initial cells and mature columella cells of the root apices, but this maximum is diminished by the presence of elevated H_2_S cells in the primary root apex (Fig. 3a). Additionally, we used [^3^H]-IAA to measure auxin transport directly. When endogenous H_2_S levels are increased by NaHS or GYY4137, the IAA movement is reduced in the root (Fig. 4). Moreover, the application of the low concentration auxin (5 nM IAA) partially removed the effects of NaHS (Fig. S6). Taken together, the evidence supports the hypothesis that H_2_S perturbs auxin distribution and transport in the root apical meristem, which inhibits the primary root elongation.

LR development is initiated by asymmetric divisions in pairs of founder cells within xylem pole pericycle cells. When the auxin levels reach a certain threshold, which is sensed and transduced by the xylem pole pericycle cells, the cell cycle machinery becomes activated, resulting in progression to the S phase^3^. Thus, the auxin maximum is required for LRP initiation^39^. Although H_2_S changes the auxin maximum distribution in the primary root apices, H_2_S does not alter the auxin maximum distribution in the entire stage of LRP development (stages I–VII) (Fig. 3d). In addition, the application of the H_2_S donor increased the density of LRP and LR (Fig. 2b & c), indicating that auxin transport may play an important role in the H_2_S-induced LR formation.

Plants can transfer IAA from source tissues to the root and shoot apices and other sink tissues, and this depends on the differential auxin transport components^7^. The PIN proteins are required for auxin transport; the PIN2 protein is a key regulator of basipetal IAA movement and the PIN1 protein regulates the acropetal IAA movement. Both PIN1 and PIN2 are important for root architecture development^39^. In the PIN family, PIN1, 3, 4 and 7 regulate LR formation^39^. The *pin1* mutant and the *pin3pin4pin7* triple mutant show strongly reduced bending of the LRs^14^,^51^. Except for *PIN3* and *PIN4*, the expression of *PIN1*, *PIN2* and *PIN7* were enhanced when the endogenous H_2_S level increased (Fig. S3). However, the IAA transport capacity was inhibited by H_2_S, suggesting that the gene expression of PIN proteins may not be a key factor in the H_2_S regulation of auxin transport. The polar subcellular distribution of PIN proteins at the plasma membrane determines the function of the PIN proteins^40^. We followed the localization of the fluorescent markers with different polar localization in the epidermal cells: the polar distribution of PIN1:GFP, PIN2:GFP, PIN4:GFP and PIN7:GFP were altered by H_2_S by changing the vesicle transport of the PIN proteins. In addition, H_2_S signaling delayed the recycling of PIN proteins between the endomembrane compartments and the PM (Fig. S8). These results reveal that the loss of polar PIN protein localization results in the H_2_S-induced decrease in the auxin transport capacity.

Vesicle trafficking of PIN proteins is an actin-dependent process^41^. The actin cytoskeleton regulates many physiological processes and controls endocytosis, exocytosis, and vesicle trafficking. Actin-dependent vesicle trafficking is also affected by signaling molecules^53^. Increases in the H_2_S levels could decrease the occupancy of F-actin bundles in root epidermic cells (Fig. 6a). In conclusion, we have demonstrated for the first time that H_2_S has the capacity to affect the percentage of F-actin. Thus, we speculate that F-actin may act as a downstream effector of H_2_S signaling and may thus affect the vesicle trafficking of PIN proteins. We further predict that it is a key regulator of cellular responses to H_2_S signaling. However, signaling-induced reorganizations or changes in the actin cytoskeleton often require ABPs as the stimulus response modulators^24^. Alternatively, H_2_S might therefore directly affect F-actin or some of the ABPs. Here, qRT-PCR analysis showed that the transcription of some of the ABPs (including *CPA*, *CPB* and *PRF3*) increased after treatment of H_2_S donor for a short time (Fig. 7b & c). GUS activities were also enhanced in the CPA::GUS, CPB::GUS and PRF3::GUS transgenic lines following H_2_S treatment (Fig. 8 & 9). In addition, the effects of H_2_S on PIN2 immunolocalization were partially weakened in the *cpb* mutants (Fig. 7S). Taken together, these data imply that H_2_S might primarily affect ABPs and then alter the morphology of actin cytoskeleton, therefore acting as the secondary effect response to H_2_S exposure. To confirm this hypothesis, we observed the effects of H_2_S on F-actin in T-DNA insertion mutants of *cpa*, *cpb* and *prf3*. Our observations indicated that the effects of H_2_S on F-actin were partially removed in the *cpa*, *cpb* and *prf3* mutant plants (Fig. 6). This finding suggests that H_2_S effectively regulates several ABPs, which are critical for the assembly and depolymerization of F-actin. Interestingly, the inhibitory role of H_2_S on auxin transport was also partially recovered compared to the WT plants in the *cpa*, *cpb* and *prf3* mutant plants (Fig. 10), suggesting that several ABPs can act as the direct regulator responses to H_2_S signaling on actin-dependent auxin transport.

In conclusion, our results provide a new insight into how H_2_S triggers changes in auxin transport and distribution in Arabidopsis roots. In the present study, we have provided evidence that high levels of H_2_S will inhibit auxin distribution and transport and will lead to alterations of root system development, including alterations in the primary root, LP and root hair. Our study suggests that the H_2_S-inhibited auxin transport in the Arabidopsis roots is due to alterations in the assembly and depolymerization of F-actin. In this process, the ABPs act as downstream effectors of H_2_S signal transduction, which regulate depolymerization of F-actin in the root cells. Finally, the distribution and transport of auxin are altered. To our knowledge, this is the first molecular evidence that H_2_S signaling regulates the remodeling of root system architecture by affecting auxin transport and actin cytoskeleton development in Arabidopsis. The regulative mechanism of H_2_S signaling on plant growth is very complicated. Our data suggest the existence of a tightly regulated intertwined signaling network between auxin, H_2_S and ABPs that is responsible for controlling root system development. Auxin can also affect the patterning and organization of the actin cytoskeleton during cell growth^6^,^54^. On the other hand, the actin cytoskeleton partially affects the directional transport of auxin by modulating the cycling of auxin efflux carriers^44^,^55^. The effects of H_2_S on root growth occur through a complex process. Alterations in the distribution may further affect its own transport and actin cytoskeleton. Thus, further research is required for the elucidation of the detailed molecular mechanisms involved in H_2_S-induced auxin homeostasis changes in plants.

## Methods

### Plant material and chemical treatments

This study was carried out on *Arabidopsis thaliana* including WT ecotypes Columbia (Col-0), the transgenic lines PIN1:GFP, PIN2:GFP, PIN4:GFP, PIN7:GFP, DR5::GUS, CPA::GUS, CPB::GUS, PRF3::GUS and the *cpa*, *cpb and prf3* mutants. Seeds were surface sterilized with 70% ethanol for 30 s and 15% sodium hypochlorite for 15 min, and washed five times with sterilized water before sowing on solid 1/2 Murashige and Skoog (MS) medium (pH 5.7) containing 1% (w/v) sucrose, 0.8% (w/v) agar. After that, the seeds were vernalized for 2 d at 4°C. Then the seedlings were grown in a growth room, which has the temperature at 22 ± 1°C and with a 14/10 h light/dark photoperiod under a photon flux density of 120 μmol m^−2^s^−1^. The Arabidopsis plants used throughout this work were grown routinely in a growth chamber under 50–60% humidity.

Following 3 or 5 d growth, Arabidopsis seedlings were transferred to the 1/2 MS agar medium. 50–1000 μM sodium hydrosulfide (NaHS), 100 μM p-(methoxyphenyl) morpholino-phosphin-odithioic acid (GYY4137), 10 μM N-1-naphthylphthalamic acid (NPA) were added to the medium for various treatments. The H_2_S donors NaHS and GYY4137, and NPA were purchased from Sigma.

### Measurement of H_2_S content

Hydrogen sulfide quantification was performed as described by Nashef^56^. Briefly, the root of seedlings were ground into fine powder with a mortar and pestle under liquid nitrogen and 0.3 g of frozen tissue were homogenized in 1 ml of 100 mM potassium phosphate buffer (pH 7.0) containing 10 mM EDTA. The homogenate was centrifuged at 15,000 *g* for 20 min at 4°C and 100 μl of the supernatant was used for the quantification of H_2_S, in an assay mixture containing also 1880 μl extraction buffer and 20 μl of 20 mM 5,5′-dithiobis (2-nitrobenzoic acid), in a total volume of 2 ml. The assay mixture was incubated at room temperature for 2 min and the absorbance was read at 412 nm. Hydrogen sulfide was quantified based on a standard curve of known concentrations of NaHS.

### Morphology measurements

After transferring the 3-d-old seedlings to various treatments for 2 or 4 d, we analyzed the number of primary roots, the LRP and the LR with a dissecting microscope. At least 25 roots were analyzed per replicate. The number of LRs (longer than 0.5 mm in the length of LR) was counted. LRPs were classified and counted according to their stage of development using the methods and nomenclature described in Malamy and Benfey^39^. LRs that had emerged but were shorter than 0.5 mm were classified as LRPs, and the density of LRPs was determined by counting the number per seedling. For each treatment, at least 25 seedlings were used for the morphology measurements. These experiments were repeated three times. Root hairs were photographed with an Olympus stereo microscope and the number of root hairs was counted in a 2 mm region from the primary root apex. The length of the primary roots, LRs and root hairs were measured with NIH Image software (Image J, version 1.43).

### Confocal microscopy and fluorescence intensity analysis

We used PIN1:GFP, PIN2:GFP, PIN4:GFP and PIN7:GFP to analyze the localizations of PIN1, PIN2, PIN4 and PIN7 in Arabidopsis ecotype Columbia (Col-0). The 5-d-old seedlings were used to observe the subcellular localization. For the various chemical treatments, 5-d-old seedlings were transferred to the 1/2 MS agar medium, which contained different chemicals, and were treated for 6 h. Fluorescent images were obtained using the Olympus DP72 laser confocal scanning microscope. For the GFP fluorescence observations, we used argon laser excitation at 488 nm with a 505–550 nm emission filter set. The anti-PIN1 antibody and the anti-PIN2 antibody from Sigma-Aldrich were diluted by 1:300. Secondary antibodies were diluted by 1:1000. To obtain the shown views, we scanned for another 2.5 μm after seeing the PIN-GFP proteins; thickness of every slice was 0.5 μm. Image J software was used to analyze the green fluorescence intensity.

### Actin Staining of root and confocal microscopy observation

To analyze the dynamic F-actin networks, F-actin was stained as previously described with slight modifications^57^. 5-d-old seedlings isolated from the WT and mutant plants were prefixed for 20 min in 1% stationary liquid, which was freshly prepared from 1% paraformaldehyde and 0.025% glutaraldehyde in PME buffer (100 mM PIPES, 5 mM MgSO_4_, 10 mM EGTA, pH 6.8). Then, the seedlings were immersed in 2% paraformaldehyde and 0.05% glutaraldehyde in PME for 20 min. Finally, the seedlings were fixed in a final concentration of 4% paraformaldehyde and 0.1% glutaraldehyde in PME for 20 min. After three washes in PME, the seedlings were stained using 0.3 μM Alexa 488-phalloidin (Molecular Probes) diluted in PME buffer with 5% dimethyl sulfoxide and 0.05% NP-40 overnight in the dark. Fluorescent images were captured using a confocal laser scanning microscope (Olympus DP72) equipped with a 409 objective. The GFP fluorescence images were collected using a 488 nm excitation laser line and a 505-530 nm band pass emission filter. To measure the amount of F-actin in the root cells, the images were captured under the same conditions. All images were analyzed using Image J software and the amount of F-actin was calculated by measuring the pixel intensity of the individual cells^58^.

### Histochemical analyses

GUS activity analysis employed a histochemical assay, which was performed as described with minor modifications^59^. Histochemical analysis was completed in 5-d-old DR5::GUS, CPA::GUS, CPB::GUS and PRF3::GUS seedlings. Seedlings were transferred to 1/2 MS agar medium containing different chemicals and treated for 6 h. Then, the samples were collected and used for the following assays. Seedlings were incubated in GUS-staining buffer containing 1 mM X-Gluc, 0.5 mM potassium ferricyanide, 100 mM sodium phosphate (pH 7.5), 0.5 mM potassium ferrocyanide, 10 mM EDTA and 0.1% Triton X-100. Tissues were incubated at 37°C for 6 h and then fixed with 70% (v/v) ethanol (n ≥ 25). Samples were photographed with the dissecting microscope.

### Quantitative GUS activity assay

The substrate 4-methylumbelliferyl-β-d-glucuronide (MUG) (Sigma) was used to assay the GUS activity^59^. Roots of the seedlings were frozen in liquid nitrogen and ground in a 300 μl MUG extraction buffer composed of sodium phosphate (50 mM, pH 7.0), 10 mM EDTA (pH 8.0), 10 mM β-mercaptoethanol, 0.1% (v/v) Triton X-100 and 0.1% (w/v) N-lauroyl sarcosine (SLS) (Sigma). The extract was spun, and the supernatant was extracted. 10 μl of the extract was mixed with 390 μl of the GUS assay buffer and incubated at 37°C for 1 h. The samples were stopped with 0.2 M Na_2_CO_3_. Fluorescence was determined by DyNA Quant 200. The protein concentration was measured according to Peterson's^60^ modification of the Lowry method. Approximately 40 seedlings were subjected to each treatment.

### Auxin transport assays

Acropetal auxin transport was measured in the root system, as described by Buer and Muday^61^, while Basipetal auxin transport was measured in the root system, as described by Shin^13^. The following modifications were applied to these methods. Here, 1 mm diameter agar blocks containing 7.76 × 108 M [^3^H]-IAA (PerkinElmer, USA) were applied to the root-shoot transition zone. After treatment with NaHS, GYY4137 and NPA for 24 h, a 0.5 mm section of the root close to the agar block was dissected and discarded. 2 mm consecutive segments below the incision line were then collected separately and pooled from 6 to 10 roots and placed in glass scintillation vials containing 5 ml scintillation solution. A Beckman Coulter LS6500 Scintillation Counter (Fullerton, CA, USA) was used to measure the radioactivity in these two pools of root segments. The radioactivity values are reported as means ± standard deviation from three independent experiments. The observed effects were corroborated by a NPA block assay^4^.

### RNA isolation and qRT-PCR

Roots of Col-0 were harvested to extract total RNA for real-time PCR. Total RNA was extracted using RNAprep pure plant kit (Tiangen, Beijing) and treated with RNase free DNase (Tiangen). The total RNA was reverse-transcribed into first-strand cDNA using PrimeScript™ Reverse Transcriptase (Takara, Japan) and Oligo (dT)_15_ primer (Takara) following the manufacturer's instructions. The samples were amplified using SYBR Green I (SYBR® Premix Ex Taq™ Kit, Takara). The housekeeping gene *EF1A* was used as an internal control. The thermal cycle used was as follows: 95°C for 10 s, and 40 cycles of 95°C for 5 s and 59°C for 25 s. This was followed by 80 cycles of 10 s during the time elapsed during 55–95°C. The PCR amplifications for each gene were performed in triplicate. The results were analyzed by Rotor-Gene Real-Time Analysis Software 6.1 (Build 81). All the primers used in this study were shown in Table S1.

### Statistical analysis

Each experiment was repeated at least three times and three replications in each time. Values were expressed as means ± SE. For all experiments, theoverall data were statistically analyzed in the SPSS version 17.0 (SPSS). Duncan's multiple range tests were used. The statistical analysis of two groups was performed using Student's *t*-test. In all cases, the confidence coefficient was set at 0.05.

## Author Contributions

J.S.L. and H.L.J. designed research; J.S.L. and H.L.J. performed research; T.T.F. and Y.F.H. constructed transgenic plants; J.S.L., H.L.J., T.T.F. and Y.F.H. analyzed data; and J.S.L. and H.L.J. wrote the paper.

## Supplementary Material

Supplementary Information Supplemental Data

## References

[b1] Woodward A. W. & Bartel B. Auxin: regulation, action, and interaction. Ann. Bot-London 95, 707–35 (2005). 10.1093/aob/mci083 PMC4246732 15749753

[b2] Normanly J. Approaching cellular and molecular resolution of auxin biosynthesis and metabolism. Cold Spring Harbor Perspectives in Biology 2, 001594–001601 (2010). 10.1101/cshperspect.a001594 PMC2827909 20182605

[b3] Benková E. *et al.* Local, efflux-dependent auxin gradients as a common module for plant organ formation. Cell 115, 591–602 (2003). 14651850 10.1016/s0092-8674(03)00924-3

[b4] Rashotte A. M., Brady S. R., Reed R. C., Ante S. J. & Muday G. K. Basipetal auxin transport is required for gravitropism in roots of Arabidopsis. Plant Physiol. 122, 481–490 (2000). 10677441 10.1104/pp.122.2.481 PMC58885

[b5] Dubrovsky J. G. *et al.* Auxin minimum defines a developmental window for lateral root initiation. New Phytol. 191, 970–983 (2011). 21569034 10.1111/j.1469-8137.2011.03757.x

[b6] Rahman A. *et al.* Auxin, actin and growth of the Arabidopsis thaliana primary root. Plant J. 50, 514–528 (2007). 17419848 10.1111/j.1365-313X.2007.03068.x

[b7] Vanneste S. & Friml J. Auxin: a trigger for change in plant development. Cell 136, 1005–1016 (2009). 19303845 10.1016/j.cell.2009.03.001

[b8] Kleine-Vehn J., Dhonukshe P., Swarup R., Bennett M. & Friml J. Subcellular trafficking of the Arabidopsis auxin influx carrier AUX1 uses a novel pathway distinct from PIN1. Plant Cell 18, 3171–3181 (2006). 17114355 10.1105/tpc.106.042770 PMC1693951

[b9] Yang Y., Hammes U. Z., Taylor C. G. & Schachtman D. P. Nielsen E high-affinity auxin transport by the AUX1 influx carrier protein. Curr. Biol. 6, 1123–1127 (2006). 16677815 10.1016/j.cub.2006.04.029

[b10] Santelia D. *et al.* MDR-like ABC transporter AtPGP4 is involved in auxin-mediated lateral root and root hair development. FEBS Lett. 579, 5399–5406 (2005). 16198350 10.1016/j.febslet.2005.08.061

[b11] Friml J. *et al.* Efflux-dependent auxin gradients establish the apicalebasal axis of Arabidopsis. Nature 426, 147–153 (2003). 14614497 10.1038/nature02085

[b12] Blilou I. *et al.* The PIN auxin efflux facilitator network controls growth and patterning in Arabidopsis roots. Nature 433, 39–44 (2005). 15635403 10.1038/nature03184

[b13] Shin H. *et al.* Complex regulation of Arabidopsis AGR1/PIN2-mediated root gravitropic response and basipetal auxin transport by cantharidin-sensitive protein phosphatases. Plant J. 42, 188–200 (2005). 15807782 10.1111/j.1365-313X.2005.02369.x

[b14] Rosquete M. R. *et al.* An auxin transport mechanism restricts positive orthogravitropism in lateral roots. Curr. Biol. 23, 817–822 (2013). 23583551 10.1016/j.cub.2013.03.064

[b15] Li J. S. & Jia H. L. cGMP modulates Arabidopsis lateral root formation through regulation of polar auxin transport. Plant Physiol. & Biochem. 66, 105–117 (2013). 23500713 10.1016/j.plaphy.2013.02.014

[b16] Dubrovsky J. G. *et al.* Auxin acts as a local morphogenetic trigger to specify lateral root founder cells. Proc. Natl. Acad. Sci. USA 105, 8790–8794 (2008). 18559858 10.1073/pnas.0712307105 PMC2438385

[b17] Geldner N. *et al.* Partial loss-of-function alleles reveal a role for GNOM in auxin transport-related, post-embryonic development of Arabidopsis. Development 131, 389–400 (2004). 14681187 10.1242/dev.00926

[b18] Hussey P. J., Ketelaar T. & Deeks M. J. Control of the actin cytoskeleton in plant cell growth. Annu. Rev. Plant Biol. 57, 109–125 (2006). 16669757 10.1146/annurev.arplant.57.032905.105206

[b19] Kandasamy M. K., McKinney E. C. & Meaghe R. B. A single vegetative actin isovariant overexpressed under the control of multiple regulatory sequences is sufficient for normal Arabidopsis development. Plant Cell 21, 701–718 (2009). 19304937 10.1105/tpc.108.061960 PMC2671709

[b20] Fan T. *et al.* Overexpression of profilin 3 affects cell elongation and F-actin organization in *Arabidopsis thaliana*. Plant Cell Rep. 32, 149–160 (2013). 23052593 10.1007/s00299-012-1349-2

[b21] Li L., Wang Y. & Shen W. Roles of hydrogen sulfide and nitric oxide in the alleviation of cadmium-induced oxidative damage in alfalfa seedling roots. Biometals 25, 617–631 (2012). 22538639 10.1007/s10534-012-9551-9

[b22] Jia H. *et al.* Arabidopsis CROLIN1, a novel plant actin-binding protein, functions in cross-linking and stabilizing actin filaments. J. Biol. Chem. 288, 32277–32288 (2013). 24072702 10.1074/jbc.M113.483594 PMC3820865

[b23] Huang S., Blanchoin L., Kovar D. R. & Staiger C. J. Arabidopsis capping protein (AtCP) is a heterodimer that regulates assembly at the barbed ends of actin filaments. J. Bio. Chem. 278, 44832–44842 (2003). 12947123 10.1074/jbc.M306670200

[b24] Huang S., Gao L., Blanchoin L. & Staiger C. J. Heterodimeric capping protein from Arabidopsis is regulated by phosphatidic acid. Mol. Bio. Cell 17, 1946–1958 (2006). 16436516 10.1091/mbc.E05-09-0840 PMC1415281

[b25] Nagai Y., Tsugane M., Oka J. & Kimura H. Hydrogen sulfide induces calcium waves in astrocytes. FASEB J. 18, 557–559 (2004). 14734631 10.1096/fj.03-1052fje

[b26] García-Mata C. & Lamattina L. Hydrogen sulfide, a novel gasotransmitter involved in guard cell signalling. New Phytol. 188, 977–984 (2010). 20831717 10.1111/j.1469-8137.2010.03465.x

[b27] Tan B. H., Wong P. T. H. & Bian J. S. Hydrogen sulfide: a novel signaling molecule in the central nervous system. Neurochem. Int. 56, 3–10 (2010). 19703504 10.1016/j.neuint.2009.08.008

[b28] Wilson L. G., Bressan R. A. & Filner P. Light-dependent emission of hydrogen sulfide from plants. Plant Physiol. 61, 184–189 (1978). 16660257 10.1104/pp.61.2.184 PMC1091829

[b29] Christou A., Manganaris G. A., Papadopoulos I. & Fotopoulos V. Hydrogen sulfide induces systemic tolerance to salinity and non-ionic osmotic stress in strawberry plants through modification of reactive species biosynthesis and transcriptional regulation of multiple defence pathways. J. Exp. Bot. 647, 1953–1966 (2013). 23567865 10.1093/jxb/ert055 PMC3638822

[b30] Li J. S., Jia H. L., Wang J., Cao Q. & Wen Z. Hydrogen sulfide is involved in maintaining ion homeostasis via regulating plasma membrane Na^+^/H^+^antiporter system in the hydrogen peroxide-dependent manner in salt-stress *Arabidopsis thaliana* root. Protoplasma 251, 899–912 (2014). 24318675 10.1007/s00709-013-0592-x

[b31] Li Z. G., Yang S. Z., Long W. B., Yang G. X. & Shen Z. Z. Hydrogen sulfide may be a novel downstream signal molecule in nitric oxide-induced heat tolerance of maize (*Zea mays* L.) seedlings. Plant Cell and Environ. 36, 1564–1572 (2013). 10.1111/pce.12092 23489239

[b32] Chen J. *et al.* Hydrogen sulfide alleviates aluminum toxicity in barley seedlings. Plant Soil 362, 301–318 (2013).

[b33] Zhang H. *et al.* Hydrogen sulfide promotes wheat seed germination and alleviates the oxidative damage against copper stress. J. Integr. Plant Biol. 50, 1518–1529 (2008). 19093970 10.1111/j.1744-7909.2008.00769.x

[b34] Dooley F. D., Nair S. P. & Ward P. D. Increased growth and germination success in plants following hydrogen sulfide administration. Plos one 8, 62048–62052 (2013). 10.1371/journal.pone.0062048 PMC3629089 23614010

[b35] Zhang H. *et al.* Hydrogen Sulfide Promotes Root Organogenesis in *Ipomoea batatas*, *Salix matsudana* and *Glycine max*. J. Integ.r Plant Biol. 51, 1086–1094 (2009). 10.1111/j.1744-7909.2009.00885.x 20021556

[b36] Fang T., Cao Z., Lia Z., Shen W. & Huang L. Auxin-induced hydrogen sulfide generation is involved in lateral root formation in tomato. Plant Physiol. & Biochem. 76, 44–51 (2014). 24463534 10.1016/j.plaphy.2013.12.024

[b37] Fernández-Marcos M., Sanz L., Lewis D. R., Muday G. K. & Lorenzo O. Nitric oxide causes root apical meristem defects and growth inhibition while reducing PIN-FORMED 1 (PIN1)-dependent acropetal auxin transport. Proc. Natl. Acad. Sci. USA 108, 18506–18511 (2011). 22021439 10.1073/pnas.1108644108 PMC3215072

[b38] Malamy J. E. & Benfey P. N. Organization and cell differentiation in lateral roots of *Arabidopsis thaliana*. Development 124, 33–44 (1997). 9006065 10.1242/dev.124.1.33

[b39] Péret B. *et al.* Arabidopsis lateral root development: an emerging story. Trends Plant Sci. 14, 399–408 (2009). 19559642 10.1016/j.tplants.2009.05.002

[b40] Wiśniewska J. *et al.* Polar PIN localization directs auxin flow in plants. Science 312, 883 (2006). 16601151 10.1126/science.1121356

[b41] Grunewald W. & Friml J. The march of the PINs: Developmental plasticity by dynamic polar targeting in plant cells. EMBO J. 29, 2700–2714 (2010). 20717140 10.1038/emboj.2010.181 PMC2924653

[b42] Staiger C. J. Signaling to the actin cytoskeleton in plants. Annual Review of Plant Physiology and Plant Mol. Biol. 51, 257–288 (2000). 10.1146/annurev.arplant.51.1.257 15012193

[b43] Dhonukshe P. *et al.* Auxin transport inhibitors impair vesicle motility and actin cytoskeleton dynamics in diverse eukaryotes. Proc. Natl. Acad. Sci. USA 105, 4489–4494 (2008). 18337510 10.1073/pnas.0711414105 PMC2393819

[b44] Mudaya G. K. & Murphy A. S. An emerging model of auxin transport regulation. Plant Cell 14, 293–299 (2002). 11884675 10.1105/tpc.140230 PMC543400

[b45] Meagher R. B. & Williamson R. E. The plant cytoskeleton. In Arabidopsis, Meyerowitz E, Somerville C, eds. Cold Spring Harbor Laboratory, Cold Spring Harbor, NY, pp 1049–1084 (1994).

[b46] Pollard T. D. & Cooper J. A. Actin, a central player in cell shape and movement. Science 326, 1208–1212 (2009). 19965462 10.1126/science.1175862 PMC3677050

[b47] Pavlov D., Muhlrad A., Cooper J., Wear M. & Reisler E. Actin filament severing by cofilin. J. Mol. Biol. 365, 1350–1358 (2007). 17134718 10.1016/j.jmb.2006.10.102 PMC2572264

[b48] Finkel T., Theriot J. A., Dise K. R., Tomaselli G. F. & Goldschmidt-Clermont P. J. Dynamic actin structures stabilized by profilins. Proc. Natl. Acad. Sci. USA 91, 1510–1514 (1994). 8108438 10.1073/pnas.91.4.1510 PMC43189

[b49] Wang J. *et al.* Arabidopsis actin capping protein (AtCP) subunits have different expression patterns, and downregulation of AtCPB confers increased thermotolerance of Arabidopsis after heat shock stress. Plant Sci. 193–194, 110–119 (2012). 10.1016/j.plantsci.2012.06.002 22794924

[b50] Wang Y. *et al.* Hydrogen sulfide enhances alfalfa (*Medicago sativa*) tolerance against salinity during seed germination by nitric oxide pathway. Plant Soil 351, 107–119 (2012).

[b51] Sabatini S. *et al.* An auxin-dependent distal organizer of pattern and polarity in the Arabidopsis root. Cell 99, 463–472 (1999). 10589675 10.1016/s0092-8674(00)81535-4

[b52] Petersson S. V. *et al.* An auxin gradient and maximum in the Arabidopsis root apex shown by high-resolution cell-specific analysis of IAA distribution and synthesis. Plant Cell 21, 1659–1668 (2009). 19491238 10.1105/tpc.109.066480 PMC2714926

[b53] Kasprowicz A., Szuba A., Volkmann D., Baluška F. & Wojtaszek P. Nitric oxide modulates dynamic actin cytoskeleton and vesicle trafficking in a cell type-specific manner in root apices. J. Exp. Bot. 60, 1605–1617 (2009). 19261922 10.1093/jxb/erp033 PMC2671617

[b54] Lanza M. *et al.* Role of actin cytoskeleton in brassinosteroid signaling and in its integration with the auxin response in plants. Dev. Cell 22, 1275–1285 (2012). 22698285 10.1016/j.devcel.2012.04.008

[b55] Sun H., Basu S., Brady S. R., Luciano R. L. & Muday G. K. Interactions between auxin transport and the actin cytoskeleton in developmental polarity of Fucus distichus embryos in response to light and gravity. Plant Physiol. 135, 266–278 (2004). 15122028 10.1104/pp.103.034900 PMC429370

[b56] Nashef A. S., Osuga D. T. & Feeney R. E. Determination of hydrogen sulfide with 5,5 β′-dithiobis-(2-nitrobenzoic acid), N-ethylmaleimide, and parachloromercuribenzoate. Analytical Biochem. 79, 394–405 (1977). 10.1016/0003-2697(77)90413-4 869184

[b57] Nishimura T., Yokota E., Wada T., Shimmen T. & Okada K. An Arabidopsis ACT2 dominant-negative mutation, which disturbs F-actin polymerization, reveals its distinctive function in root development. Plant Cell Physiol. 44, 1131–1140 (2003). 14634149 10.1093/pcp/pcg158

[b58] Zhang H. *et al.* Arabidopsis VILLIN5, an actin filament bundling and severing protein, is necessary for normal pollen tube growth. Plant Cell 22, 2749–2767 (2010). 20807879 10.1105/tpc.110.076257 PMC2947167

[b59] Jefferson R. A., Kavanagh T. A. & Bevan M. W. GUS fusions: beta-glucuronidase as a sensitive and versatile gene fusion marker in higher plants. EMBO J. 6, 3901–3907 (1987). 3327686 10.1002/j.1460-2075.1987.tb02730.x PMC553867

[b60] Peterson G. L. A simplification of the protein assay method of Lowry et al. which is more generally applicable. Analytical Biochem. 83, 346–356 (1977). 10.1016/0003-2697(77)90043-4 603028

[b61] Buer C. S. & Muday G. K. The transparent testa4 mutation prevents flavonoid synthesis and alters auxin transport and the response of Arabidopsis roots to gravity and light. Plant Cell 16, 1191–1205 (2004). 15100399 10.1105/tpc.020313 PMC423209

