# Supplementary material for: Hydrogen sulfide modulates actin-dependent auxin transport via regulating ABPs results in changing of root development in Arabidopsis
Source: Sci Rep. 2015 Feb 5;5:8251. doi: 10.1038/srep08251 (PMC4317700; doi:10.1038/srep08251)
Supplement: Supplementary Information — Supplemental Data [file srep08251-s1.pdf]

Title:

Hydrogen sulfide modulates actin-dependent auxin transport via regulating ABPs results in changing of root development in Arabidopsis

Honglei Jia[a], Yanfeng Hu[b], Tingting Fan[c], Jisheng Li[a*]

[a]College of Life Sciences, Northwest A&F University, Yangling, Shaanxi 712100, China

[b]Key Laboratory of Mollisols Agroecology, Northeast Institute of Geography and Agroecology, Chinese Academy of Sciences, Nangang District, Harbin 150000, China

[c]School of Biotechnology and Food Engineering, Hefei University of Technology, Hefei, Anhui 230009, China

*Author to whom correspondence should be addressed: Jisheng Li

Fax: +86 029 87092262, E-mail: lijsh2011@163.com

Supplemental data

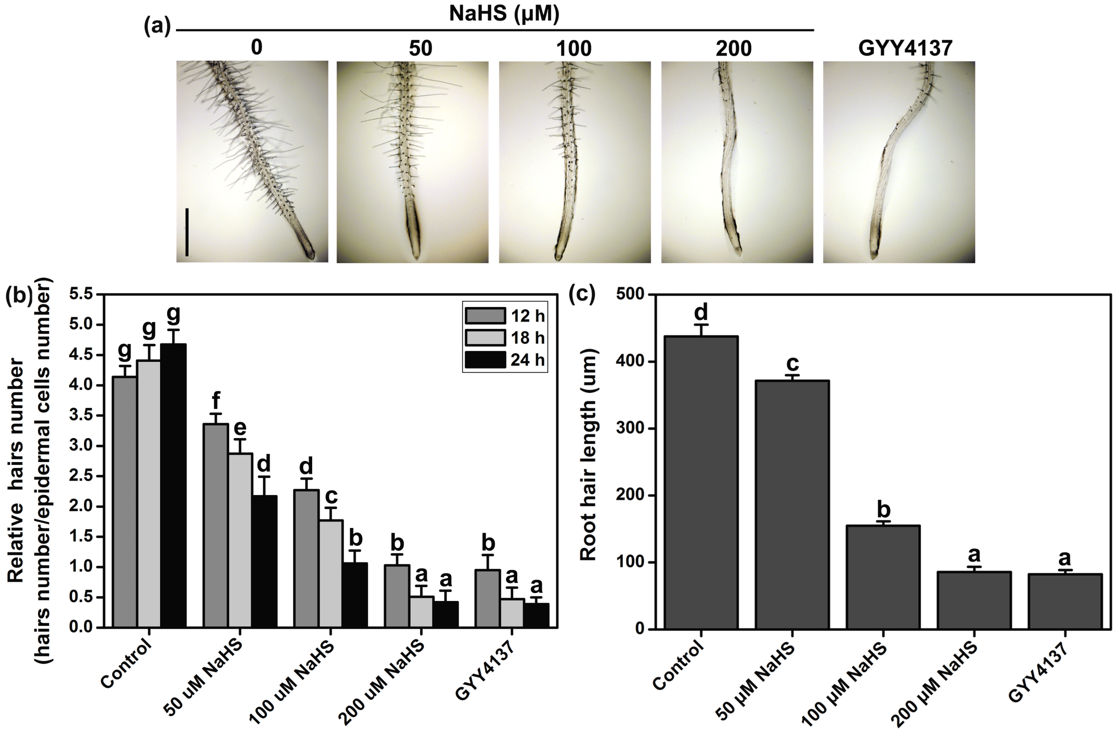

Figure S1 Effects of H_2S on root hair development in WT seedlings. (a) Photographs of root hairs formed under various treatments for 18 h in the primary root tip. Images shown are representative of each treatment. Scale bar = 500 μm. (b) The number of root hairs were counted in a 2 mm region from the primary root apex under various treatments for 12-24 h. To remove the effect of cell length, we determined the epidermal cell length and the number of cells in the 2-mm section, then calculating the relative root hairs number (root hairs number/epidermal cells number). (c) Root hair length were counted under various treatments for 18 h. The 5-d-old seedlings were transferred onto vertical plates containing 50-200 μM NaHS or 100 μM GYY4130 for the various treatments. Data are mean values and SE (n > 25). Within each set of experiments, bars with different letters are significantly different ($P < 0.05$, Duncan's multiple range tests).

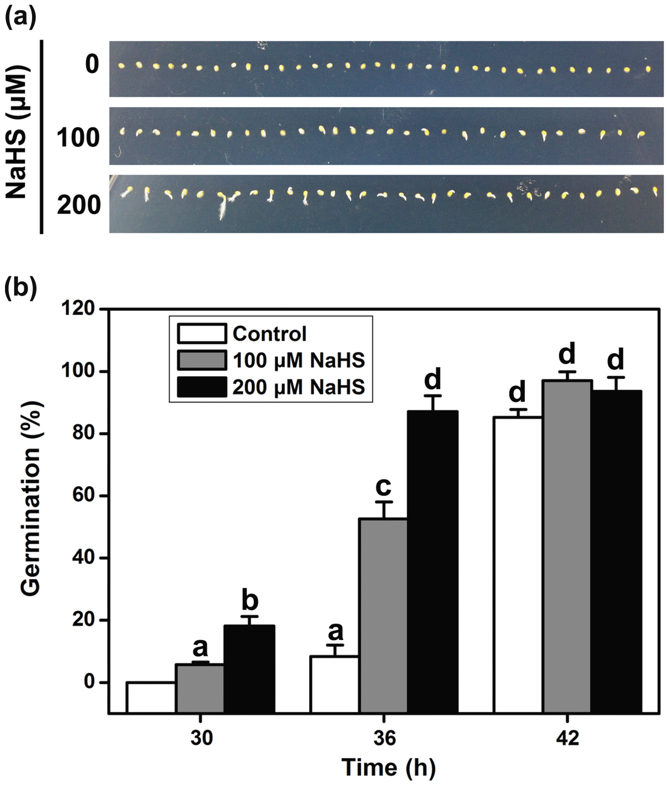

Figure S2 Effects of H_2S on germination of WT Arabidopsis seeds. (a) Representative photographs of seeds germination in 100 and 200 μM NaHS treatments for 36 h. (b) Germination Rate of WT seeds were counted under 100 and 200 μM NaHS for 30-42 h. Data are mean values and SE (n = 100). Within each set of experiments, bars with different letters are significantly different ($P < 0.05$, Duncan's multiple range tests).

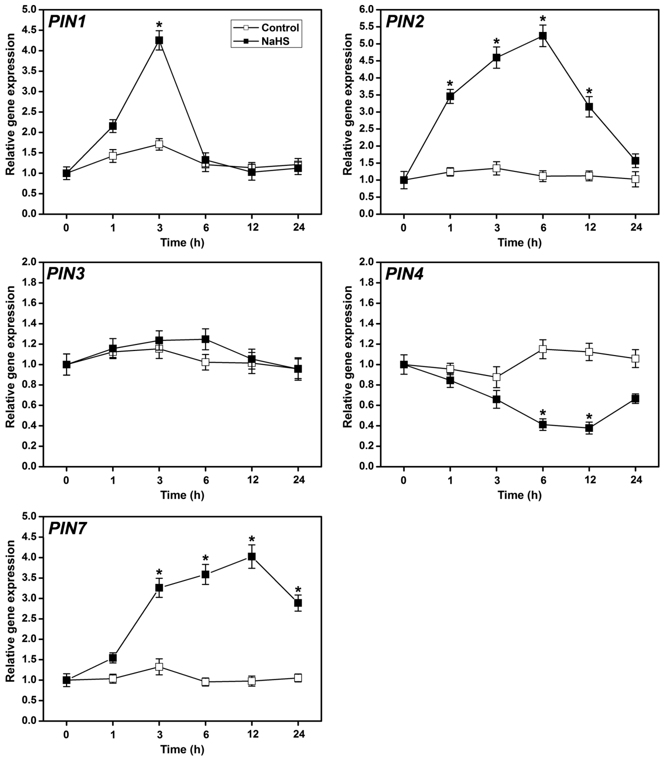

Figure S3 qRT–PCR analysis of *PINs* genes in WT Arabidopsis root. Relative expression levels are normalized to *EF1a*. 5-d-old seedlings were grown on agar plates supplied with 200 μM NaHS for 1-24 h. Mean values and SE are calculated from three replicates. Within each set of experiments, bars with different letters are significantly different at the $P < 0.05$ level (Duncan's multiple range tests).

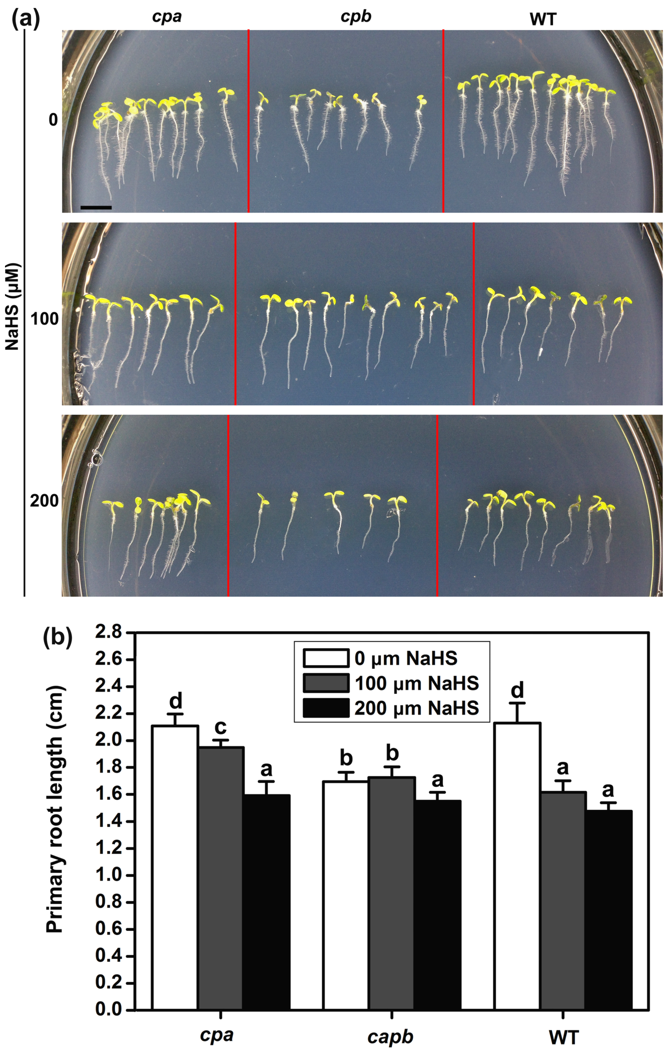

Figure S4 Effects of H_2S on the regulation of primary root growth in mutant plants. (a) Photograph showing the length of primary root of WT, *cpa* and *cpb* mutant plants seedlings. 3-d-old seedlings were transferred to 1/2 MS agar plates grown for 3 d. The agar plate were untreated (Control) or supplemented with 100 or 200 µM NaHS. Scale bar = 1cm. (b) The length of primary root were obtained 3 d after the treatment of 3-d-old seedlings. Data are mean values and SE (n > 25) in (a) and (b). Within each set of experiments, bars with different letters are significantly different ($P < 0.05$, Duncan's multiple range tests).

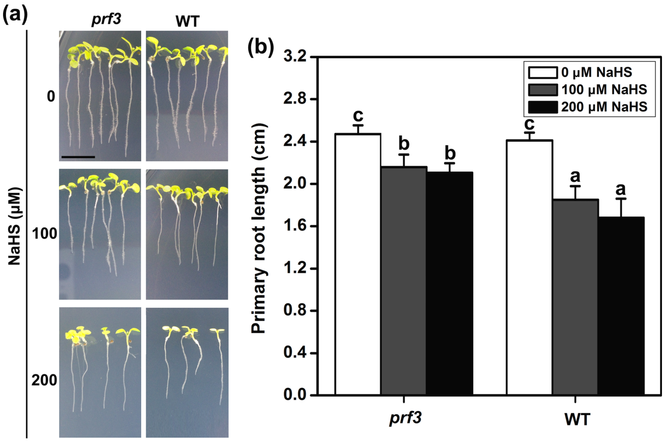

Figure S5 Effects of H_2S on the regulation of primary root growth in *prf3* mutant plants. (a) Photograph showing the length of primary root of WT and *prf3* mutant plants seedlings. 3-d-old seedlings were transferred to 1/2 MS agar plates grown for 3 d. The agar plate were untreated (Control) or supplemented with 100 or 200 μM NaHS. Scale bar = 1cm. (b) The length of primary root were obtained 3 d after the treatment of 3-d-old seedlings. Data are mean values and SE ($n > 25$) in (a) and (b). Within each set of experiments, bars with different letters are significantly different ($P < 0.05$, Duncan's multiple range tests).

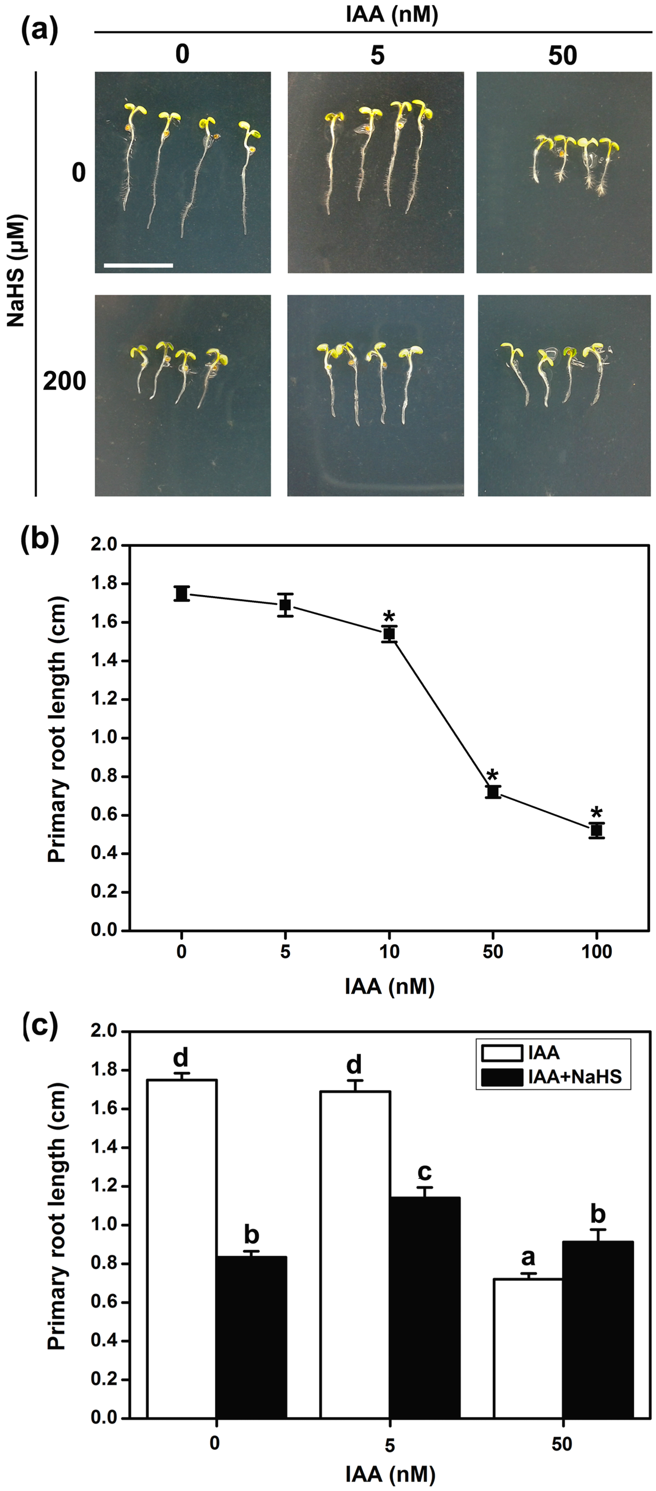

Figure S6 Effects of IAA on H_2S-inhibited primary root elongation in WT seedlings. 5-d-old seedlings treated with 0-200 µM NaHS and 0-100 nM IAA for 2 d. Images shown are representative of each treatment (a). Scale bar = 10 cm. Different concentration of IAA affcted primary root elongation (b). Effects of IAA and NaHS on primary root elongation (c). Data are mean values and SE (n > 25) in (b) and (c). Within each set of experiments, bars with different letters are significantly different ($P < 0.05$, Duncan's multiple range tests and Student's *t* test).

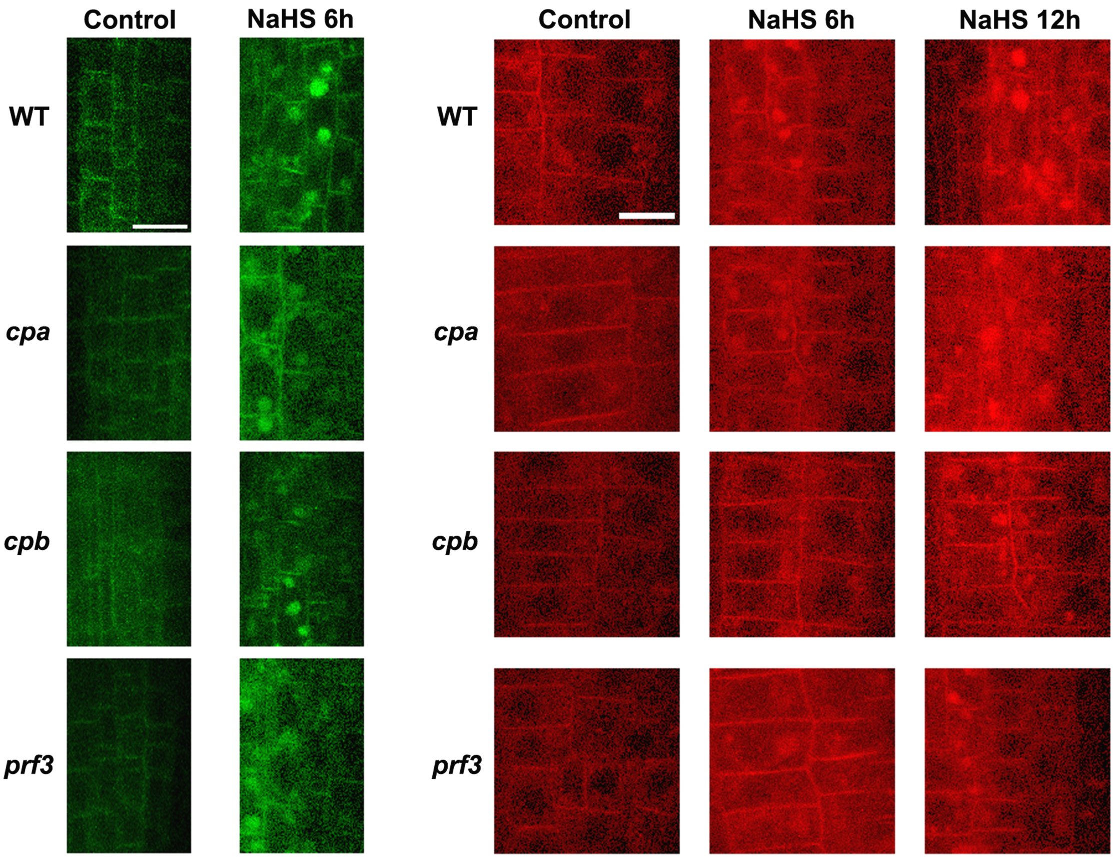

Figure S7 The effect of H_2S the PINs localization in the *cpa*, *cpb*, *pfr3* mutants and WT. Immunolocalisztion of PIN1 (a) and PIN2 (b) were imaged. 5-d-old seedlings treated with 200 μM NaHS for 6 and 12 h. Images shown are representative of each treatment. Scale bar = 10 μm.

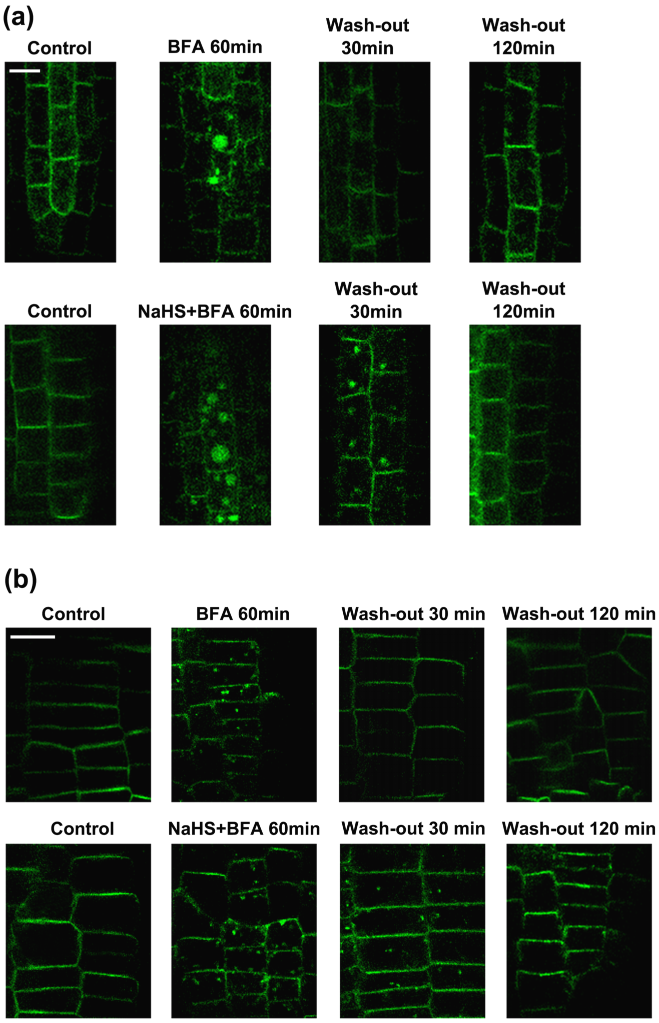

Fugrue S8 The effect of H_2S on recycling of PIN1 and PIN2 from BFA compartments to plasma membrane. PIN1:GFP (a) or PIN2:GFP (b) were imaged. 5-d-old seedlings treated with 200 μM NaHS and 20 μM BFA for 60 min, then NaHS and BFA wash-out after 30 and 120 min. Images shown are representative of each treatment. Scale bar = 10 μm.

TABLE S1 Primers and accession numbers used in this study

Primer name	accession number	Sequence (5'- 3')
PIN1 F	AT1G73590	CGGTGGGAACAACATAAGCA
PIN1 R		GGTGATGCTGAATAAACTGGA
PIN2 F	AT5G57090	CCGTGGGGTTAAGCTTCTCATCT
PIN2 R		AGCTTTCCGTCGTCTCGTATCTCC
PIN3 F	AT1G70940	TCTTTGATTAGGTTCTGGTAACTC
PIN3 R		GCTCATGTGAAACTGCAACAAG
PIN4 F	AT2G01420	CAACAGAGGGATCCCCATGACA
PIN4 R		GCCTTCGAATCTTACCGGAGCT
PIN7 F	AT1G23080	TCCACTTCATCTCCTCAAACAATC
PIN7 R		TGTGATGCTCTATTCAAGACTACC
CPA F	AT3G05520	CTATGGCGGACGAAGAAGATG
CPA R		CGGGAGCGTTGAGGAAAA
CPB F	AT1G71790	CTTTGCGATCTATCGTGACCAG
CPB R		TGACCTGACTTTGACCCATCTTT
PRF1 F	AT2G19760	ATCFCCTTTAGTGTGATCT
PRF1 R		TTTTTCGCCACCGAGAAA
PRF2 F	AT4G29350	ACCATCTCATGTGCGAGG
PRF2 R		CTTCTCACCCCCAAGGAT
PRF3 F	AT5G56600	ATGTTGCAGGCAACCGCCTC
PRF3 R		TGGGGCAAGTGTTCCAGGTGT
ADF1 F	AT3G46010	TGGATCTCGATGTTTTCAGG
ADF1 R		AGATCTTTCAAGGGGATGATGA
ADF2 F	AT3G46000	TGTGTTATGTGTGCCTTGTGA
ADF2 R		TTGAAGCCTAGCAACAATCTGA
ADF3 F	AT5G59880	TGTGACGGTACCAACCTGAA
ADF3 R		GGGTTCGATTTCTT CACAA
ADF4 F	AT5G59890	CACCCTTCATTCTATCTGTCTGG
ADF4 R		CAAAGGAAGCAAACACAGCA

ADF6 F	AT2G31200	TGTTGATGAGCATGATGAGAGA
ADF6 R		TGTTCGCAAGGATTAGACTCG
ADF11 F	AT1G01750	TTGGAGTTAAAAGCAAAGAGG
ADF11 R		TTTGAGCTTGCATACAACAT
EF1a F	AT1G18070	ACCACGAGTCTCTTCTTGAGGCAC
EF1a R		TGGCAGGGTCATCCTTGGAG

The "(F)" indicates forward primer and "(R)" indicates reverse primer.
